# Triangulating Instrumental Variable, confounder adjustment and difference-in-difference methods for comparative effectiveness research in observational data

**DOI:** 10.12688/wellcomeopenres.22955.1

**Published:** 2025-02-10

**Authors:** Laura M. Güdemann, John M. Dennis, Andrew P. McGovern, Lauren R. Rodgers, Beverley M. Shields, William Henley, Jack Bowden, on behalf of the MASTERMIND consortium

**Affiliations:** 1 Faculty of Health and Life Sciences, University of Exeter, Exeter, Devon, EX2 5DW, UK

**Keywords:** causal inference, unmeasured confounding, triangulation, Instrumental Variable method, Prior Event Rate Ratio approach

## Abstract

**Background:**

Observational studies play an important role in assessing the comparative effectiveness of competing treatments. In clinical trials the randomization of participants to treatment and control groups generally results in balanced groups with respect to possible confounders, which makes the analysis straightforward. However, when analysing observational data, the potential for unmeasured confounding makes comparing treatment effects more challenging.

**Methods:**

Causal inference methods such as Instrumental Variable and Prior Event Rate Ratio approaches make it possible to circumvent the need to adjust for confounding factors that have not been measured in the data or measured with error. Direct confounder adjustment via multivariable regression and propensity score matching also have considerable utility. Each method relies on a different set of assumptions and leverages different aspects of the data.

The assumptions of each method are described, and the impact of their violation is assessed in a simulation study. We propose the prior outcome augmented Instrumental Variable method that leverages data from before and after treatment initiation and is robust to key assumption violations. Finally, we propose a heterogeneity statistic to decide if two or more estimates are statistically similar, considering their correlation. We illustrate our framework in an application study assessing the risk of genital infection in type 2 diabetes patients prescribed SGLT2-inhibitors versus DPP4-inhibitors using UK primary care data.

**Results:**

Our proposed approach can estimate treatment effects without bias in scenarios where assumptions of other methods are violated. Furthermore, the application study exemplified the usefulness of discussing the consistency of estimation results from different estimation methods using triangulation.

**Conclusion:**

Triangulating results of different estimation methods is important in observational data to derive high quality evidence. The proposed triangulation framework and heterogeneity statistic are valuable tools to discuss the consistency of estimation results from different methods to shed light on possible sources of bias.

## Plain Language Summary

Not all research questions and patient populations can be assessed in clinical trials due to ethical issues, costs, and practicality considerations. Therefore, it is important for medical research to derive high-quality real world evidence from observational data, which has been collected for purposes such as administrative tasks and not primarily for research. To assess and compare the effect of treatment options on a specific health outcome of interest from observational data, suitable statistical methods need to be employed to address data quality issues. Ideally, all factors that can simultaneously influence the treatment decision of a clinician and the health outcome of interest need to be included in the analysis to control for their effects and accurately analyse the treatment effect of interest. If some of these factors are not measured in the data, advanced statistical methods need to be employed. We introduce a new method to address this issue and compare it with existing methods. Additionally, we illustrate all methods discussed in a case study for type 2 diabetes research. Our analysis shows that our proposed method is able to provide high quality observational evidence in certain scenarios. Furthermore, the comparison of different methods and our outlined case study provides insights into strengths and weaknesses, of the methods which are important considerations when choosing between them for observational research.

## 1 Introduction

The gold standard approach for evaluating the efficacy of treatments is a randomized controlled trial (RCT). Due to strict specifications of RCTs with regard to blinding and randomization of treatment assignment, causal conclusions about the treatment’s effect on patient outcomes can be drawn without the need to adjust for prognostic factors, since they should be well-balanced across trial arms. This remains true even if the trial is affected by non-adherence, and non-adherence is predicted by the aforementioned prognostic variables ^1^.

Observational data, for example from electronic healthcare records, provide vital means for assessing the comparative effectiveness of commonly prescribed medications with similar indications. Since these data are collected as part of routine care, treatment assignment is not randomized. This opens up the possibility that treatment choice (or the extent of treatment received) and patient outcomes may be simultaneously predicted by common variables, which could bias the analysis because of a lack of balance across treatment groups, in contrast to the adherence affected RCT setting previously discussed. This phenomenon is referred to as ‘confounding’ and we will refer to such common factors as confounders from now on ^1– 3^.

Standard causal inference methods such as stratification, multivariable regression or propensity score matching make it possible to analyse observational data and draw causal conclusions as long as all confounders can be accurately measured and appropriately adjusted for ^1^. For example, Dawwas *et al.* ^4^ used propensity score matched data for a retrospective cohort study for a comparative risk analysis of cardiovascular outcomes in people with type 2 diabetes (T2D) initiating Dipeptidyl peptidase-4 inhibitors (DPP4i) versus Sodium-glucose co-transporter-2 inhibitors (SGLT2i) therapy. Another example using standard causal inference methods is McGovern *et al.* ^5^ who used multivariable Cox regression and propensity scores to define important clinical groups of people with T2D initiating either DPP4i or SGLT2i, with high risk of genital infection.

Failure to measure and appropriately adjust for all confounders can bias the estimation of the true causal effect of treatment on the outcome of interest. Two causal inference approaches which circumvent the problem of unmeasured confounding are the Instrumental Variable (IV) and the Prior Event Rate Ratio (PERR) method. The IV approach addresses confounding by substituting each patients’ observed treatment with a predicted treatment. This prediction is made using a variable (the instrument) that is assumed to be independent of any confounders and only affects the outcome through the treatment. Randomization to a treatment group within a RCT is perhaps the best example of an IV, and can therefore be used to adjust for non-adherence ^1, 6, 7^. Because of this, IV analyses using observational data are generally equated with the creation of a pseudo-randomized controlled trial.

Examples of IVs for observational data include geographic information such as the distance to the nearest health facility ^8^, germ line genetic information ^9^ or healthcare providers’ preference for a particular treatment ^10^. In this paper we will subsequently construct an IV of this latter type.

The Prior Event Rate Ratio method ^11^ is an alternative quasi-experimental approach which leverages data at two time points. Specifically, the outcome must be measured in the ‘prior’ period before initiation of treatment and then in the ‘study’ period after treatment has commenced. The treatment effect is first estimated in the prior period by (somewhat paradoxically) regressing the prior outcome on the study period treatment indicator. This is assumed to capture the degree of unmeasured confounding in the treatment effect subsequently estimated in the study period, which can then be subtracted out to de-bias the analysis. The approach relies on the assumption of time invariant unmeasured confounding across both time periods. For related reasons it is necessary that a patient’s outcome in the prior period does not directly influence the allocation of the study period treatment. Furthermore, the prior and study event of interest should be of the same nature and non-terminal, such as death ^11, 12^. The PERR method is generally applied to time-to-event data but is directly analogous to the method of difference-in-difference (DiD) regression in the case of continuous or binary outcomes.

**Figure 1.**
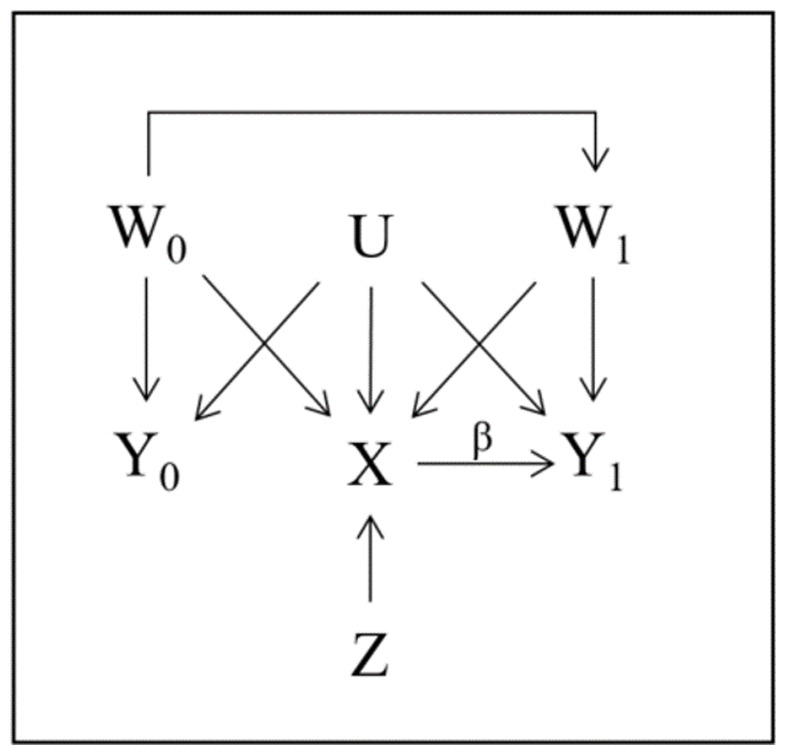
Causal diagram showing the relationship between *Y*_0_, *Y*_1_, *X*, *U*, *W*_0_ and *W*_1_ in the case where both the IV and DiD assumptions are satisfied. The estimates and assumptions are explained in detail in Section 2.

Figure 1 shows a causal diagram illustrating the possible relationship between: the outcome in the prior and study periods ( *Y*_0_ and *Y*_1_ respectively); the treatment indicator ( *X*); an IV ( *Z*); measured confounders of treatment and outcome in both periods ( *W*_0_ and *W*_1_ respectively); and unmeasured confounders ( *U*). In this diagram, the assumptions related to variable relationships of both the DiD (analogous to PERR) and IV approaches are satisfied, but the ‘no unmeasured confounder’ assumption underlying a direct adjustment strategy is not.

In this paper we consider the joint application of direct confounder adjustment, IV and DiD approaches for estimating the causal effect of treatment using observational data. In Section 2 we give a more detailed description of each method and introduce a heterogeneity statistic to decide if two or more estimators are sufficiently similar. In Section 3 we assess the performance of these approaches in a detailed simulation study. In Section 4 we consider an extension of the standard IV approach using pre- and post-treatment outcome data that can be used in scenarios where the assumptions of both the standard IV and DiD approaches are violated. We call this extension the prior outcome augmented Instrumental Variable method (POA-IV). In Section 5 we apply our methods to routinely collected healthcare records to assess the causal effect of SGLT2i compared to DPP4i as second-line therapy on the risk of genital infections, exploiting variation in healthcare provider prescribing habits to construct an IV. We conclude in Section 6 with a discussion and point to further research. All source codes for this research including for all simulations and the application study as well as supplementary material in form of an appendix are available online at https://github.com/GuedemannLaura/POA-IV and at Güdemann *et al.* ^13, 14^.

## 2 Methods

We are interested in estimating the comparative effectiveness of two treatments ( *X* = 1 compared to *X* = 0) on outcome *Y*_1_ using observational data. Using the potential outcome notation, let *Y*_1 *i*_( *X _i_ = x*) denote the outcome of patient *i* if assigned treatment level *X _i_ = x*. The target of this analysis is a hypothetical estimand:

$$\;\beta =E[Y_{1i}(X_{i}=1)]-E[Y_{1i}(X_{i}=0)].\;(1)$$

That is, the difference in expected outcomes if all patients could receive treatment level 1 compared to treatment level 0. For simplicity, we will assume in this section that the outcome of interest is continuous, with the extension to binary outcomes discussed in Section 2.4.

### 2.1 The ‘as Treated’ and ‘Corrected as Treated’ estimate

In a RCT with complete adherence to the assigned treatment, hypothetical estimand β could be consistently estimated using the ‘ *as Treated*’ estimate, by comparing the average outcome across both treatment groups:

$${\hat{\beta }}_{aT}=\hat{E}[Y_{1}\text{|}X=1]-\hat{E}[Y_{1}\text{|}X=0].$$

Complete adherence could be illustrated in Figure 1 by letting *Z* represent the randomized treatment assignment and removing all arrows into *X* from *W*_0_, *W*_1_ and *U*, so that only the *Z* → *X* arrow remains. Difficulties emerge when calculating the *as Treated* estimate with observational data, because treatment assignment is not randomized or controlled by the researcher. It is then possible that factors exist which simultaneously affect (or confound) the treatment assignment and the outcome. This would lead to an imbalance across the treatment groups with respect to *W*_0_, *W*_1_ and *U* and the estimate ${\hat{\beta }}_{aT}$ will consequently be biased due to confounding.

If all confounding factors are known and can be appropriately measured and adjusted for - which we call the ‘no unmeasured confounder’ (NUC) assumption - a ‘ *Corrected as Treated*’ (CaT) estimate that is additionally adjusted for these factors can consistently estimate β.

Returning to Figure 1, if the NUC assumption held so that *U* was absent from the diagram it would be sufficient to adjust for *W*_1_ since *W*_0_ only affects *Y*_1_ through *W*_1_ and the CaT estimate would be

$$\;{\hat{\beta }}_{CaT}=\hat{E}[Y_{1}\text{|}X=1,W_{1}]-\hat{E}[Y_{1}\text{|}X=0,W_{1}].\;(2)$$

This could be estimated from fitting the following multivariable regression of Y _1_ on X and W _1_ as

$$\text{E}[{\text{Y}}_{1}|\text{X},{\text{W}}_{1}]=\beta_{0}+\beta_{\text{CaT}}\text{X}+\beta_{1}{\text{W}}_{1}.$$

### 2.2 The Instrumental Variable estimate

In many settings the NUC assumption may be thought unreasonably strong. The Instrumental Variable (IV) method offers a means for circumventing the problem of unmeasured confounding to consistently estimate the hypothetical estimand. It works via the construction of a pseudo-randomized controlled trial using a variable *Z* which needs to fulfill the following three assumptions in order to be a valid IV:

IV1: *Z* is associated, or predicts *X*;
IV2: *Z* is independent of *Y*_1_ given *W*_0_, *W*_1_, *X* and *U*;
IV3: *Z* and *Y*_1_ do not share a common cause.

IV1 is often referred to as the relevance assumption and the *Z - X* relationship can be empirically tested from a regression of *X* on *Z*. The assumption would be invalidated if this association is weak, with an *F*-statistic of at least 10 often used as a threshold for good strength of an IV ^15^. Assumption IV2 is also referred to as the exclusion restriction and requires *Z* to only influence *Y*_1_ through *X* but not directly. IV3, the exchangeability assumption, requires that *Z* and *Y*_1_ are not themselves confounded ^16, 17^.

In its simplest form where only one IV is used and no adjustment for covariates is made, the IV estimate for β is the ratio of the *Y*_1_ – *Z* association and the *X* – *Z* association:

β^IV=E^[Y1|Z=1]−E^[Y1|Z=0]E^[X|Z=1]−E^[X|Z=0](3)

In order to enable consistent estimation of hypothetical estimand ( 1) using ( 3), we additionally make the homogeneity assumption that the average treatment effect is constant across both levels of the IV *Z*, at each level of the treatment ^7^:

$$E[Y_{1i}(X=1)-Y_{1i}(X=0)\text{|}Z=1,X=x]=E[Y_{1}(X=1)-Y_{1}(X=0)\text{|}Z=0,X=x].$$

A more general method for IV estimation with a continuous outcome that allows for multiple IVs and covariate adjustment is Two-Stage Least Squares (TSLS) ^16^. To implement TSLS with a single IV *Z* and the measured confounder *W*_1_, we first fit a logistic regression model for *X* given *Z* and *W*_1_:

$$\;\text{Logit}\left(P(X=1\text{|}Z,W_{1})\right)=\alpha_{X,0}+\alpha_{X,Z}Z+\alpha_{X,W_{1}}W_{1}\;(4)$$

The estimated coefficients of this model are then used to predict *X* given *Z* and *W*_1_ as $\hat{X},$ which is then itself regressed on *Y*_1_ and *W*_1_ in a second-stage model:

$$\;E\left(Y_{1}\text{|}\hat{X},W_{1}\right)=\alpha_{Y_{1,}0}+\beta_{IV}\hat{X}+\alpha_{Y_{1},W_{1}}W_{1}\;(5)$$

The coefficient of $\hat{X}$ is then taken as the TSLS estimate ^17^. Using a valid IV, the TSLS is consistent under the homogeneity assumption and additionally assuming that the covariates are correctly modelled in ( 5) ^18, 19^.

### 2.3 Difference-in-difference estimate

An alternative approach to adjust for unmeasured confounding is the difference-in-difference (DiD) estimate. It can be applied to continuous and binary outcomes and is conceptually equivalent to the Prior Event Rate Ratio (PERR) method typically applied to time-to-event outcomes ^6^. Borrowing the terminology of the PERR approach, DiD estimation leverages data from two periods: the *prior* period before drug initiation and the *study* period after drug initiation. For the estimation of the treatment effect in the study period, the treatment effect measured for the prior period is used to capture the degree of unmeasured confounding. The method presumes that the treatment effect measured in the prior period reflects the composite effect of measured and unmeasured confounders on the outcome, if none of the participants receive any of the study treatments in the prior period ^11, 12, 20, 21^. Once estimated, it can then be subtracted from the X→Y _1_ association (or the *as Treated* estimate). This approach relies on the following assumptions:

- DiD1: *Y*_0_ does not influence the treatment decision *X* directly
- DiD2: The effect of *U* on the outcome is constant across time conditional on *W*_0_ and *W*_1_^11, 12^.

Previous studies show that the DiD method is biased in case of the violation of assumption DiD1 ^22, 23^ and DiD2 ^24^. A formal proof that these assumptions are sufficient for identification of hypothetical estimand ( 1) is given in Appendix 2 (Extended data). In Figure 1 assumption DiD1 is satisfied and throughout all presented studies we assume that DiD2 is satisfied.

For continuous outcomes the DiD estimate can be calculated by subtracting the results of two linear regressions from the prior and study period:

$$\;\begin{array}{c} {\hat{\beta }}_{DiD}=\hat{E}[Y_{1}\text{|}X=1,W_{1}]-\hat{E}[Y_{1}\text{|}X=0,W_{1}]\\ \;-(\hat{E}[Y_{0}\text{|}X=1,W_{0}]-\hat{E}[Y_{0}\text{|}X=0,W_{0}]). \end{array}\;(6)$$

The DiD estimate can also be calculated for a sample of *n* individuals via the following single regression model:

$$\;E[Y^{*}\text{|}X^{*},W^{*},P^{*}]=\gamma_{0}+\gamma_{P^{*}}P^{*}+\gamma_{X^{*}}X^{*}+\beta_{DiD}P^{*}\cdot X^{*}+\gamma_{W^{*}}W^{*}+\gamma_{W^{*}P^{*}}W^{*}\cdot P^{*}.\;(7)$$

Here, X* ∈ {0, 1} and P ^*^ ∈ {0, 1} are 2n-length treatment and period indicator variables and the variables Y* = (Y _0_, Y _1_) ^⊤^, W* = (W _0_, W _1_) ^⊤^ summarize the information of outcomes and covariates for both periods in a vector of the same size. The regression coefficients of the P* · X* interaction term is taken as the DiD estimate ^25^. Fitting this model facilitates the easy extraction of a standard error for the DiD estimate directly from the hessian matrix.

The DiD method utilizes only two outcome measurements before and after treatment initiation and can be viewed as a simple special case of an interrupted time series analysis, which incorporates data from multiple time points within a formal longitudinal model ^26– 29^. Due to the limitation of our own data on outcome measurements and our focus on triangulating findings across methods, we restrict our attention to the DiD approach in this paper.

### 2.4 Extension to binary outcomes

In case of a binary outcome the comparative treatment effect can be estimated using the CaT, IV, CF and DiD models using logit or probit models, instead of linear regressions for continuous outcomes. Whilst TSLS is the standard tool for IV analysis with continuous outcomes, the control function (CF) method is typically used for binary outcomes. This method can accommodate linear and non-linear associations between the IV and treatment and between the treatment and the outcome ^16, 30^. For the first stage model and continuous outcomes the IV is regressed on treatment and all measured confounders, as shown in model ( 4). From this regression the residual $\hat{\Delta }=X-\hat{X}$ model is calculated and used in the second stage model with

$$\;\text{Logit}\left(P(Y_{1}=1\text{|}X,Z,W_{1},\hat{\Delta })\right)=\beta_{Y_{1},0}+\beta_{Y_{1,}W_{1}}W_{1}+\beta_{CF}X+(\beta_{Y_{1},\hat{\Delta }}+\beta_{Y_{1},Z\hat{\Delta }}Z)\hat{\Delta }.\;(8)$$

We will use both the standard IV and CF approaches for IV analyses going forward.

When employing logistic regression models for the CaT, IV, CF and DiD models, for our purposes we prefer to extract the treatment estimate as a risk difference, or an Average Marginal Effect (AME) ^7^. For example, in the case of the CF estimate, after fitting model ( 8) to obtain estimates for its constituent parameters, the AME is calculated as the difference in average predicted probabilities when *X* is fixed at 1 and 0 respectively:

$${\hat{\beta }}_{CF}=\frac{1}{n}\sum_{i=1}^{n}\left\{\hat{P}(Y_{1}=1\text{|}X=1,Z,W_{1},\hat{\Delta })-\hat{P}(Y_{1}=1\text{|}X=0,Z,W_{1},\hat{\Delta })\right\}$$

In R, this can easily be done using the margins() package ^31^. Choosing a scale that is collapsible makes it more straightforward to compare estimates across different methods which use different covariate adjustment sets. Even if their respective assumptions are all satisfied, using a non-collapsible scale such as an odds ratio could mean that the underlying causal estimands of two methods are in fact distinct ^32^.

### 2.5 Similarity statistic

In order to assess the similarity of the estimates, after taking care to estimate them on the same scale and whilst accounting for their correlation, we use a generalized heterogeneity statistic ^33^ of Cochran’s Q ^34^ of the form

$$\;Q_{e}=\left({\hat{\beta }}_{e}-{\hat{\beta }}_{IVW,e}\right){\hat{\Sigma }}_{e}^{-1}{\left({\hat{\beta }}_{e}-{\hat{\beta }}_{IVW,e}\right)}^{T}\;(9)$$

where

- *e* is the set of estimates, for example { *CaT, IV, DiD*};
-
${\hat{\beta }}_{e}$ is a vector of all estimates in *e* with ***j*** th entry ${\hat{\beta }}_{ej}$

-
${\hat{\beta }}_{IVW,e}$ is the inverse variance weighted average of all estimates in *e*;
-
${\hat{\Sigma }}_{e}$ is the covariance matrix for ${\hat{\beta }}_{e}$ , approximated by a non-parametric bootstrap;
-
${\hat{\beta }}_{IVW,e}$ is calculated using *w _ej_* the inverse variance of the corresponding estimate and
$${\hat{\beta }}_{IVW,e}=\frac{\Sigma_{j\in e}w_{ej}{\hat{\beta }}_{ej}}{\Sigma_{j\in e}w_{ej}}.$$

Under the null hypothesis that all estimates in *e* are targeting the same underlying quantity, Q _e_ is asymptotically $\chi_{{\text{n}}_{\text{e}}-1}^{2}$ distributed, *n _e_* indicating the number of estimates in the set *e*. This hypothesis is rejected at level α if ${\text{Q}}_{\text{e}}>\chi_{{\text{n}}_{\text{e}}-1}^{2}(1-\alpha )$ and the estimates are assumed not to be similar. Equation ( 9) can therefore be used to assess the extent of agreement across estimators and, by extension, the validity of the assumptions that they rest on. This approach can be seen as a generalisation of the causal triangulation framework for uncorrelated estimates described in Bowden *et al.* ^35^. We showcase an application of the *Q _e_* in Section 3.3.

## 3 Simulation study

In this section we employ a Monte-Carlo simulation to study the performance of the CaT, IV (TSLS and CF) and DiD estimates in scenarios where their specific assumptions are variously satisfied and violated. The simulation was conducted in R Studio (version 4.1.2) ^36, 37^ and the set-up is motivated to a degree by the applied analysis in Section 5. All simulations are organized and reported with respect to the recommendations given in Morris *et al.* ^38^.

### 3.1 Simulation set up

Across 1000 independent simulations, observational data is generated for n = 5000 patients grouped into *n _g_* = 50 clusters, with each cluster representing a healthcare provider (e.g. a general practitioner). The full data generating models are summarized in Appendix 1 (available online, Güdemann *et al.* ^13, 14^) but the main features are now described. The treatment group indicator *X* and the outcome variables, *Y*_0_ and *Y*_1_ are simulated as binary variables, in each case representing the presence or absence of a binary adverse event. The true treatment effect, quantified on the risk difference scale is β = 0.1. Therefore, the average causal effect of treatment 1 versus 0 is a 10% increase in adverse event risk. Further information on the chosen parameter values can be found in the additional provided material at https://github.com/GuedemannLaura/POA-IV and in the Appendix published by Güdemann *et al.* ^13, 14^.

Treatment and outcome variables are allowed to depend in principle on: measured confounders, *W*_0_ and *W*_1_; one unmeasured confounder *U* (normally distributed); and the IV *Z* (simulated as a binary variable). Specifically, *Z* is constant at the healthcare provider level, and therefore conveys information about providers’ preference to prescribe one treatment over the other.

**Figure 2.**
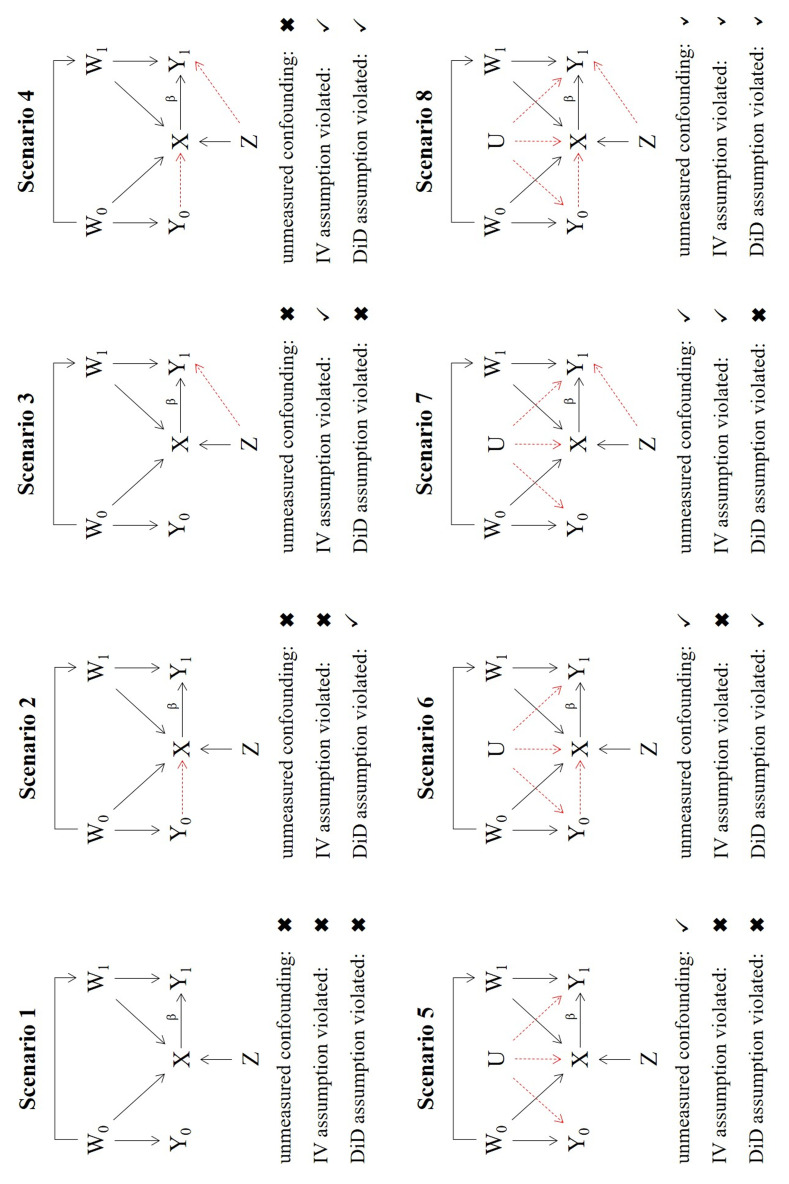
DAGs representing the scenarios of the simulation.

Figure 2 summarizes the 8 scenarios implemented in the simulation. In scenario 1, the NUC, IV and DiD assumptions are all satisfied. In scenarios 2–4, the NUC assumption is satisfied, but certain IV and DiD assumptions are violated. Specifically, in Scenario 2, the DiD1 assumption which requires *X* to be independent of *Y*_0_ is violated. This could be, for example, because the occurrence of an adverse event in the prior period influences the providers’ treatment decision in the study period. In scenario 3, IV2 (exclusion restriction) is violated. This could for example represent the case where providers’ preference for treatment is associated with their tendency to record adverse events. Previous knowledge about adverse events for a specific treatment could easily give rise to this effect. Both the IV2 and the DiD1 assumptions are violated simultaneously in scenario 4. In scenarios 5 to 8 unmeasured confounding is present (NUC violated) although it is constant over the two time periods. In addition to the unmeasured confounding, the DiD1 and IV2 assumptions are violated in scenarios 6 and 7 respectively. In Scenario 8 the NUC, IV2 and DiD1 assumptions are all violated. The eight scenarios are illustrated using causal diagrams in Figure 2. The CaT, IV, CF and DiD estimates were calculated by fitting the models listed in Table 1 using logistic regression in tandem with the margins() package ^31^, as described in the previous section. As IV2 is violated in scenarios 3, 4, 7 and 8, *Z* becomes a measured confounder of *Y*_1_. Is it therefore included in the CaT and DiD model for these scenarios.

**Table 1.** Summary of the models for CaT, IV, CF and DiD fitted in the simulation. For scenarios 3, 4, 7, 8 *Z* is included in the DiD and CaT model as measured confounder.

Estimate		Fit
CaT	${\hat{\beta }}_{\text{CaT}}$	*Y*_1_ ∼ *X* + *W*_1_
IV	${\hat{\beta }}_{IV}$	First stage model: *X* ∼ *W*_1_ + *Z* Second stage model: $Y_{1}∼\hat{X}+W_{1}$
CF	${\hat{\beta }}_{CF}$	First stage model: *X* ∼ *W*_1_ + *Z* Second stage model: $Y_{1}∼X+W_{1}+\hat{\Delta }+\hat{\Delta }\cdot Z$
DiD	${\hat{\beta }}_{DiD}$	Y* ∼ P* + X* + P* · X* + W* + W* · P*

### 3.2 Simulation results

**Figure 3.**
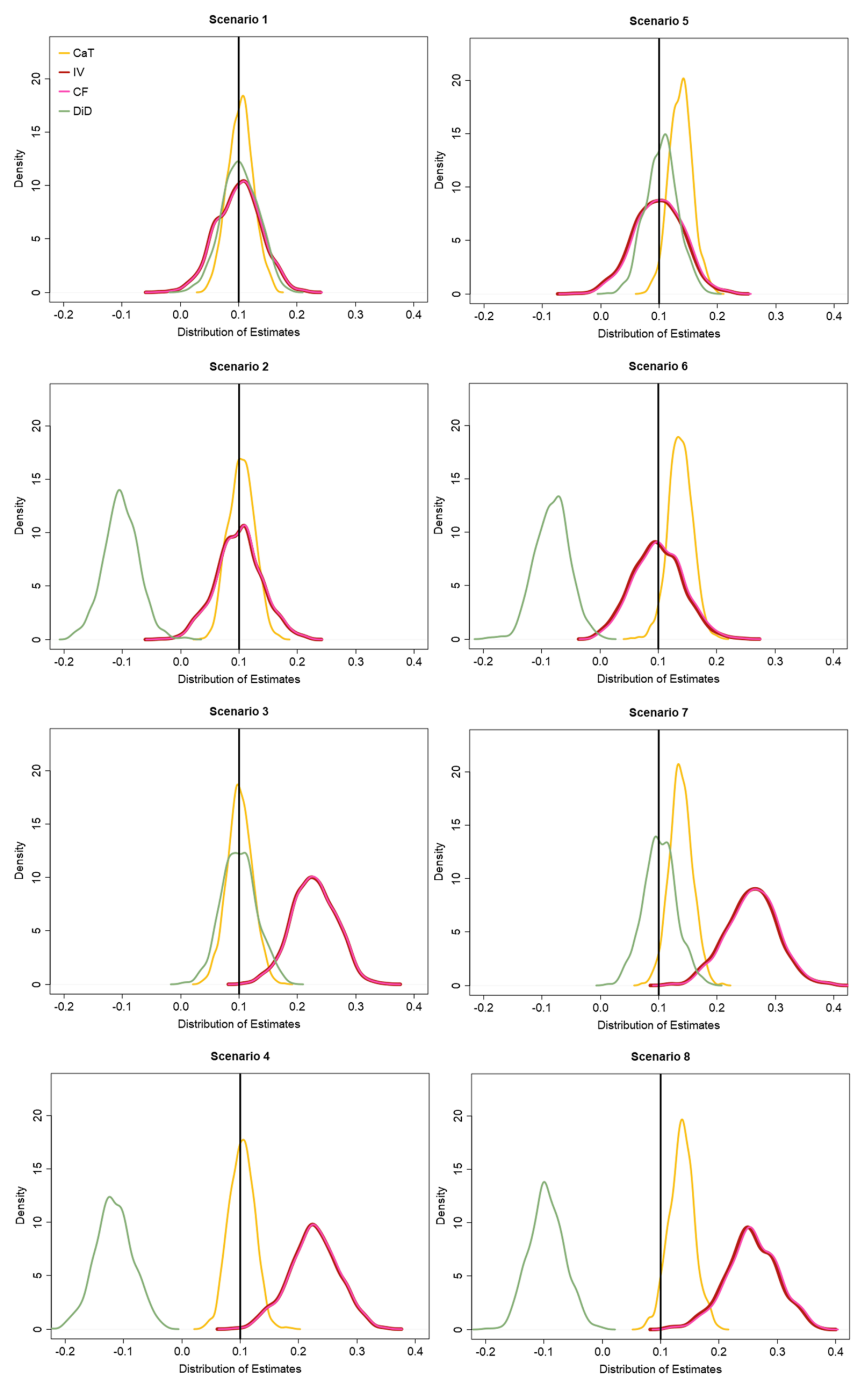
Distribution of estimation results for the CaT, IV, CF and DiD method for all simulation scenarios.

Simulation results are summarized for all 8 scenarios in Table 2. Specifically, we use the 1000 CaT, IV, CF and DiD estimates to calculate the: bias, and mean squared error (MSE); the mean empirical standard error (SE) arising directly from the model fits; coverage rate of 95% confidence intervals and the type 1 error (T1E) rate when rejecting the null hypothesis of no causal effect at the 5% significance level. In order to assess the type 1 error simulation calculations were executed with β = 0. The most efficient and unbiased method for each scenario is highlighted in Table 2 in **bold**. Figure 3 shows the distribution of the CaT, IV, CF and DiD estimates over all simulation runs. Additional simulation results including the Monte Carlo standard error estimates of the performance measures as described in Morris *et al.* ^38^, are given in Appendix 3 (Extended data).

**Table 2.** Bias, standard errors (S.E.) and mean squared error (MSE) (all × 100); coverage and type 1 error (T1E) rate (both expressed as a percentage based on a 95% confidence interval and 5% significance threshold) for the estimates CaT, IV, CF and DiD and for all scenarios.

		CaT	IV	CF	DiD
Scenario 1	bias	**0.313**	-0.012	0.062	0.251
	S.E.	**0.069**	0.121	0.121	0.099
	MSE	**0.048**	0.146	0.146	0.098
	coverage	**94.1**	95.2	95.0	93.4
	T1E	**6.0**	5.7	5.5	5.0
Scenario 2	bias	**0.411**	-0.035	0.095	-20.198
	S.E.	**0.069**	0.125	0.125	0.096
	MSE	**0.050**	0.156	0.157	4.171
	coverage	**95.0**	95.2	95.2	0.0
	T1E	**5.3**	4.8	4.8	100.0
Scenario 3	bias	**0.134**	12.801	12.879	-0.002
	S.E.	**0.069**	0.121	0.121	0.096
	MSE	**0.047**	1.784	1.805	0.092
	coverage	**94.2**	8.1	8.0	94.3
	T1E	**4.6**	94.9	94.8	6.3
Scenario 4	bias	**0.261**	12.587	12.720	-21.623
	S.E.	**0.069**	0.135	0.135	0.102
	MSE	**0.047**	1.765	1.799	4.780
	coverage	**96.4**	13.9	12.9	0.0
	T1E	**4.7**	88.3	88.4	100.0
Scenario 5	bias	3.715	-0.169	0.111	**0.436**
	S.E.	0.063	0.134	0.133	**0.087**
	MSE	0.177	0.178	0.177	**0.077**
	coverage	53.4	95.2	94.7	**95.0**
	T1E	45.4	5.9	5.9	**4.8**
Scenario 6	bias	3.771	**-0.160**	**0.184**	-18.163
	S.E.	0.065	**0.135**	**0.135**	0.094
	MSE	0.184	**0.182**	**0.182**	3.386
	coverage	55.4	**96.4**	**96.0**	0.0
	T1E	38.5	**5.6**	**5.6**	100.0
Scenario 7	bias	3.789	15.902	16.195	**0.205**
	S.E.	0.063	0.140	0.140	**0.089**
	MSE	0.184	2.724	2.818	**0.080**
	coverage	55.5	6.2	5.2	**94.6**
	T1E	45.3	96.5	96.8	**5.1**
Scenario 8	bias	3.602	15.527	15.877	-19.509
	S.E.	0.068	0.141	0.141	0.096
	MSE	0.176	2.610	2.719	3.898
	coverage	60.4	7.4	6.5	0.0
	T1E	39.4	94.8	95.1	100.0

For scenario 1, we confirm that the CaT, IV, CF and DiD estimates are all unbiased for the hypothetical estimand β, and the CaT estimate is most efficient. The coverage and T1E rates for all estimates are close to their nominal levels. In scenario 2, the DiD estimate is systematically biased and consequently has poor coverage and T1E. Since a non-zero Y _0_ → X relationship does not affect the CaT, IV or CF approaches, they estimate the treatment effect without bias, with the CaT being the most efficient. In scenario 3, which was intended to showcase the impact of IV2 assumption violation only, the IV and CF estimates are biased and their coverage/T1E rates are also adversely affected. The diagram for scenario 3 in Figure 2 reveals that due to the direct effect of *Z* on *Y*_1_, *Z* is an additional confounder, which must be included in the regression models for the CaT and DiD estimates. The results for the CaT and DiD estimates therefore include *Z* as measured confounder. Again, the CaT estimate is the most efficient in this scenario. In scenario 4 both the IV and DiD assumptions are violated and *Z* becomes a measured confounder for *Y*_1_ again. Results of the CaT and DiD estimates additionally adjusted for *Z* as a confounder show that only CaT remains unbiased in this scenario.

For scenario 5 to 8, unmeasured confounding was implemented. Consequently, the CaT estimate is biased and displays lower coverage and higher T1E compared to the IV, CF and DiD estimates, as expected. Comparing the latter two, the DiD estimate is the most efficient unbiased effect measure in scenario 5. When the DiD1 assumption is also violated in scenario 6, its estimate is again biased and shows very low coverage and high T1E rates. Only the IV and CF estimates remain unbiased. Similar to scenario 4, in scenario 7 the DiD estimate will be only biased if *Z* is not included in the model. The CaT estimate on the other hand remains biased due to the unmeasured confounding. In scenario 8, the identifying assumptions of all three methods are violated. Consequently, none of the methods are able to estimate the treatment effect without bias. This scenario may very well represent the reality of a given analysis setting. For this reason in Section 4 we discuss an extension of the standard Instrumental Variable method that can give consistent estimates for the causal effect under a different set of assumptions.

### 3.3 Similarity statistic performance

As proof of concept for the *Q _e_* statistic explained in Section 2, we repeat simulation scenario 1, 2, 3 and 5 with 500 simulation runs. In each simulation run, *Q _e_* is calculated using 500 non-parametric bootstrap samples of the observed data, with replacement. Table 3 shows the rejection rates (in %) when testing if the CaT, CF and DiD estimates are similar at the 5% level using all three pairwise comparisons.

**Table 3.** Rejection rates and 95% confidence intervals (in %). Results are shown for all pairwise combinations of the estimates.

	*e*	*H*_0_ rejected (%)	95% CI
Scenario 1	CaT, CF	7.4	5.1; 9.7
	CaT, DiD	6.4	4.3; 8.6
	CF, DiD	6.4	4.3; 8.6
Scenario 2	CaT, CF	6.0	3.9; 8.1
	CaT, DiD	100.0	100.0; 100.0
	CF, DiD	100.0	100.0; 100.0
Scenario 3	CaT, CF	96.2	94.5; 97.9
	CaT, DiD	4.4	2.6; 6.2
	CF, DiD	87.8	84.9; 90.7
Scenario 5	CaT, CF	18.2	14.8; 21.6
	CaT, DiD	40.8	36.5; 45.1
	CF, DiD	4.0	2.3; 5.7

In Scenario 1 all three estimators target the same true estimand β. Although we see a small degree of type 1 error inflation, rejection rates for each test are reassuringly low. In scenario 2 the data is generated without unmeasured confounding, but the DiD1 assumption is violated. Therefore, the DiD estimator targets a distinct estimand from the CaT or CF approaches, which themselves target the same estimand. This is indeed reflected by the test results, where we observe 100% power to detect a difference between the DiD estimate and either the CaT or CF estimates and a low power of 6% to distinguish the CaT and CF estimates themselves. In Scenario 3 (where only the CaT and DiD estimates are truly similar) and Scenario 5 (where only CF and DiD estimates are truly similar) the *Q _e_* statistic exhibits comparable performance. In scenario 5 we can only reliably detect that the DiD and CF estimates are similar but we do not have enough power to detect the differences with the CaT estimate. This might be because the bias due the violation of the NUC assumption is relatively small as well as the true difference in estimates.

## 4 The prior outcome augmented Instrumental Variable method

In this section we introduce the prior outcome augmented Instrumental Variable (POA-IV) estimate which aims to overcome the limitations of the DiD and standard IV estimate by leveraging data from both the prior and study period. Specifically, we look to leverage an interaction between the prior outcome *Y*_0_ and the original IV *Z* to form a new IV. This general technique of using interaction terms has been successfully applied in several different contexts in recent years. For example to disentangle direct and indirect effects in a mediation analysis ^39^, to allow for violation of the homogeneity assumption in a non-adherence affected RCT ^7^, and to adjust for bias due to pleiotropy in Mendelian randomization ^40^. The POA-IV estimates follow the same idea as the mentioned studies using interaction terms as instruments.

The treatment effect, β _*POA*– *IV*_ can be estimated using a slightly modified TSLS approach. In the first stage model, treatment assignment *X* is regressed on *Z* and *W*_1_, but also on *Y*_0_ and the interaction term *Y*_0_ *Z* as the new IV:

$$\;\text{Logit}\left(P(X=1\text{|}Z,W_{1},Y_{0})\right)=\alpha_{X,0}+\alpha_{X,Z}Z+\alpha_{X,W_{1}}W_{1}+\alpha_{X,Y_{0}}Y_{0}+\alpha_{X,Y_{0}Z}Y_{0}Z.\;(10)$$

By including *Y*_0_ in the first stage model, the estimate acknowledges that the treatment decision can be affected by previously measured outcomes such as drug specific adverse events or other outcomes in the prior period. Fitted values from regression model ( 10), $\hat{X},$ are then used in the second stage model:

$$\;\text{E}\left(Y_{1}\text{|}\hat{X},W_{1},Y_{0},Z\right)=\beta_{Y_{1},0}+\beta_{\text{POA}-\text{IV}}\hat{X}+\beta_{Y_{1},W_{1}}W_{1}+\beta_{Y_{1},Z}Z+\beta_{Y_{1},Y_{0}}Y_{0}\;(11)$$

to furnish a causal estimate for *X* whilst additionally controlling for any direct effects of *Z* and *Y*_0_ on *Y*_1_. The interaction term α _*X*, *Y*_0_ *Z*_ in model ( 10) would be present in our setting if a provider only shows a prescription preference in situations where the patient has already experienced an event of interest in the prior period or, more generally, if the strength of preference varies across levels of *Y*_0_. As it serves as a new IV we require that

- POA-IV1: The interaction term *α*_*X*, *Y*_0_ *Z*_ is non-zero and strong (in order to avoid weak instrument bias ^39^);
- POA-IV2: *Z* and *Y*_0_ (and hence $\hat{X}$ in model ( 11)) are independent of U.

To implement the approach using the CF model for binary outcomes (which we refer to as POA-CF), we again fit model ( 10) to the data to give $\hat{X}.$ From this we calculate the residual $\hat{\Delta }=X-\hat{X},$ and then fit the second stage regression model

$$\begin{array}{l} \text{Logit}\left(P(Y_{1}=1\text{|}X,W_{1},Y_{0},Z,\hat{\Delta })\right)=\beta_{Y_{1},0}+\beta_{POA-CF}X+\beta_{Y_{1},W_{1}}W_{1}+\beta_{Y_{1},Z}Z+\beta_{Y_{1},Y_{0}}Y_{0}\\ \;+(\beta_{Y_{1},\hat{\Delta }}+\beta_{Y_{1},Z\hat{\Delta }}Z)\hat{\Delta }, \end{array}\;(12)$$

before estimating the causal effect on the risk difference scale using the margins() package as before. The performance of both the POA-CF and equivalent standard IV method (referred to as POA-IV) are explored in the next section.

### 4.1 Simulation study

**Figure 4.**
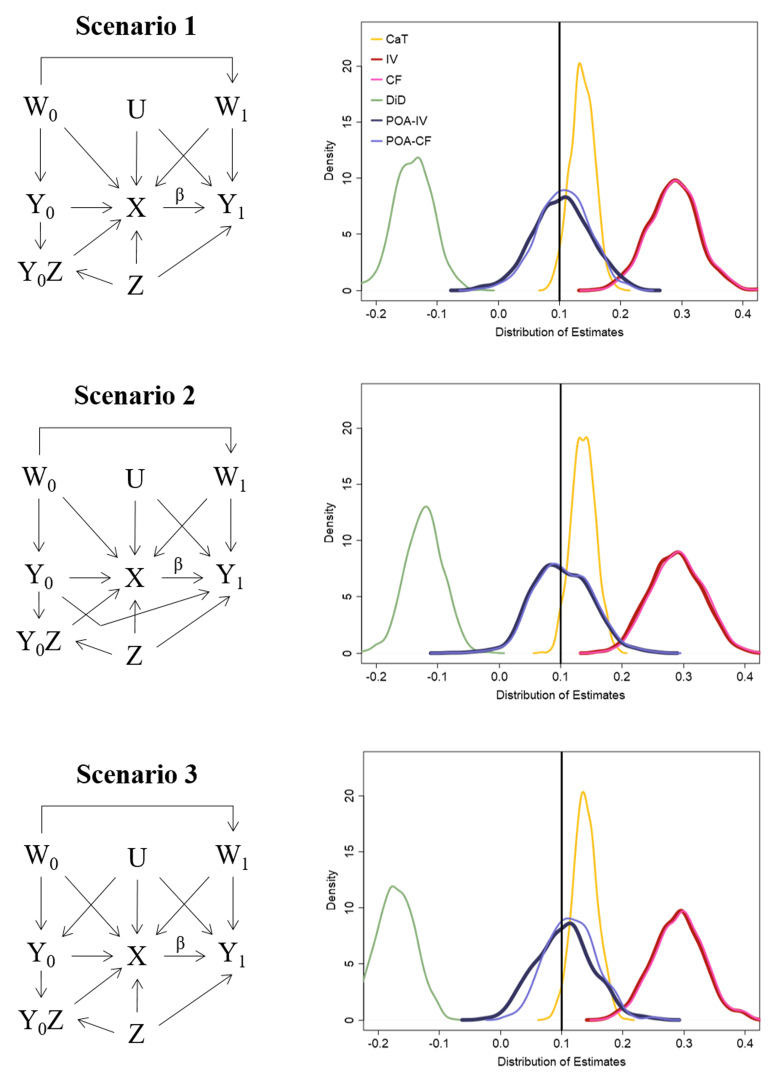
Left side: DAG representing the data generation for each scenario of the simulation. Right side: Distribution of the estimation results.

We now showcase the ability of the POA-IV and POA-CF estimate in comparison to the CaT, IV, CF and DiD estimates under conditions in which the latter three approaches are biased. The simulation is therefore an extension of the simulation described in Section 3. The left side of Figure 4 clarifies how the data for each scenario is generated. For scenario 1 and 2 of this simulation study the prior outcome *Y*_0_ is generated without unmeasured confounding. Additionally, in scenario 2 *Y*_0_ has a direct effect on the study outcome of interest *Y*_1_. Scenario 3 is the same as scenario 8 of the simulation in Section 3. Further information about the data generation models are outlined in Appendix 4 (Extended data).

### 4.2 Simulation results

The results of scenario 1 in Table 4 and the right side of Figure 4 show that the POA-IV and POA-CF are able to estimate the true causal risk difference (10%) without bias and similar efficiency to the standard IV estimate. All other methods compared in this simulation exhibit bias. This is also the case for scenario 2 in which *Y*_0_ exerts a direct effect on *Y*_1_. For this scenario *Y*_0_ was included in the outcome models as measured confounder. Coverage rates of the POA-IV and POA-CF are around 95%. The bias of the IV and CF approach in all scenarios stems from a relatively large effect of *Z* on *Y*_1_. Scenario 3 of this simulation is the same as scenario 8 described in Section 3. All previously applied methods exhibited noticeable bias. As *Y*_0_ is confounded with *U* in this scenario, POA-IV as well as POA-CF are biased too. From the results of this simulation the bias is much smaller than the bias of CaT, IV, CF and DiD, but it would increase in case of a stronger effect of U on *Y*_0_. Additional information on the Monte Carlo simulation errors are given in Appendix 5 (available online, Güdemann *et al.* ^13, 14^).

**Table 4.** Bias, standard errors (S.E.) and mean squared error (MSE) (all × 100); coverage and type 1 error (T1E) rate (both expressed as a percentage based on a 95% confidence interval and 5% significance threshold) for the estimates CaT, IV, CF, DiD, POA-IV and POA-CF and for all scenarios.

		CaT	IV	CF	DiD	POA-IV	POA-CF
Scenario 1	bias	3.718	18.579	18.875	-23.910	0.369	0.636
	S.E.	0.062	0.129	0.129	0.101	0.150	0.136
	MSE	0.176	3.617	3.729	5.819	0.225	0.188
	coverage	52.8	0.4	0.4	0.0	94.4	95.2
	T1E	44.4	99.4	99.4	100	5.9	5.9
Scenario 2	bias	3.714	18.724	19.143	-22.194	0.219	0.527
	S.E.	0.061	0.135	0.135	0.097	0.150	0.150
	MSE	0.175	3.689	3.848	5.021	0.227	0.228
	coverage	53.6	0.6	0.5	0.0	95.7	95.6
	T1E	45.7	99.3	99.4	100.0	4.0	4.2
Scenario 3	bias	3.845	18.979	19.302	-27.120	0.428	1.586
	S.E.	0.063	0.131	0.131	0.099	0.149	0.131
	MSE	0.187	3.774	3.898	7.454	0.225	0.196
	coverage	50.7	0.3	0.3	0.0	95.5	92.8
	T1E	48.9	99.7	99.7	100.0	6.0	6.2

## 5 Application to type 2 diabetes patients in Clinical Practice Research Datalink

In addition to lifestyle modification, treatment for type 2 diabetes (T2D) primarily focuses on the management of blood glucose, with different glucose-lowering oral agents available. Metformin (MFN) is recommended as first-line medical therapy by major T2D clinical guidelines ^41, 42^, but if glucose control deteriorates, additional second-line or further treatments are prescribed. Sodium-glucose co-transporter-2 inhibitors (SGLT2i) and Dipeptidyl peptidase- 4 inhibitors (DPP4i) are two widely used second-line medication classes in the UK and US ^43, 44^, and there is considerable interest in using observational data to establish the comparative benefits and risks of the two therapies in ‘real-world’ settings and for a broad spectrum of patients ^45^. Whilst SGLT2 inhibitors have some benefits beyond blood sugar lowering (including reducing the risk of cardiovascular disease) they may be associated with increased risk for genital infection ^42^.

We used routine data from the Clinical Practice Research Datalink to examine the risk of genital infections for people with T2D initiating SGLT2i (n = 1966) compared to DPP4i (n = 4033) during 2016–2019 as second-line treatment after MFN ^46, 47^. CPRD is a rich source of primary care data for observational health research. This database includes approximately 6.9% of the UK population and patients are considered to be representative with regard to age, sex and ethnicity ^46^. Studying the efficacy and tolerability with routine practice data makes it possible to understand the risks and benefits of medication use in a large and truly representative population in contrast to clinical trials which are performed on a population restricted by factors such as age or diabetes severity ^48, 49^.

All individuals in the study cohort initiated MFN as first-line treatment and have not been prescribed insulin over the complete follow-up time. Additionally, only individuals who initiated DPP4i or SGLT2i as second-line treatment were included in the analysis. The prior period is observed from start of the initiation of MFN until just before the start of the second-line treatment (SGLT2i or DPP4i). The average follow-up time in this period was 3.66 years. Therefore, the study period starts with the initiation of the second-line treatment until one of the following censoring reasons: end of follow-up data (30th of June 2019), discontinuation of second-line treatment or start of the other comparison treatment (eg. individual started to take SGLT2i as second-line treatment and added DPP4i at a later point in time). The average follow-up time of the study period was 1.44 years.

Reporting of this application study in this Section has been done with respect to the STROBE guidelines ^50^ for observational studies in Epidemiology. The baseline characteristics of the cohort are summarized in Table 5, for the two periods before and after initiation of second-line treatment. In the prior period 151 (2.5%) genital infections are recorded, 45 (2.3%) and 106 (2.6%) for people on SGTL2i and DPP4i respectively. In the study period 139 (2.3%) people experience an infection, 96 (4.9%) on SGLT2i and 43 (1.1%) on DPP4i. Genital infection is therefore a rare outcome. Data was also extracted on patients’ general practice membership, in order to use it as an IV within the standard and prior outcome augmented IV approach.

**Table 5.** Baseline data on CPRD T2D cohort for prior and study period and for patients on DPP4i (n = 4033) or SGLT2i (n = 1966) as second-line treatment. Values are shown in mean (standard deviation) unless otherwise stated.

	Prior period		Study period	
Variable	DPP4i	SGLT2i	DPP4i	SGLT2i
HbA1c (mmol/mol)	70.57 (19.30)	72.43 (19.75)	69.85 (15.27)	73.95 (16.01)
BMI (kg/m ^2^)	33.24 (6.33)	36.35 (6.80)	32.45 (6.38)	35.80 (6.70)
eGFR (ml/min/1.73 *m* ^2^)	85.03 (19.74)	92.70 (18.20)	83.18 (22.86)	93.08 (18.75)
Age (years)	61.09 (10.97)	55.26 (9.01)	65.02 (11.58)	58.61 (9.24)
T2D duration (years)	2.30 (3.01)	1.84 (2.59)	6.21 (4.36)	5.17 (3.60)
Gender				
female	41.04%	60.99%		
male	58.95%	39.01%		
Prescription year				
2016			30.17%	18.81%
2017			30.83%	31.56%
2018			28.61%	34.42%
2019			10.39%	15.21%

The outcome, defined as 1 ≥ genital infection in a given period, was coded as a binary variable and modelled using logistic regression. Causal estimates are reported on the risk difference scale (in %) as described in Section 2.4.

For our analysis we applied the six causal estimation strategies introduced in Section 2 and Section 4 to estimate the population averaged effect of taking SGLT2i versus DPP4i on infection risk. Additionally, we applied the CaT estimator on a propensity score matched dataset (PMS) using the R package MatchIt() ^51^. with 1–1 nearest neighbour matching. Approximately two-thirds of the data was matched. The balance diagnostic statistic are summarized with a love-plot in Appendix 6 (Extended data) and show that the matching procedure has improved the bal ance of the treatment groups. This plot also gives a list of the variables which was used for the matching procedure. Furthermore, the CaT and DiD method were applied including *Z* as measured confounder to avoid bias in case the exclusion restriction of the IV method is not met, which cannot be verified with the data at hand ^17^. *Y*_0_ was included as a measured confounder in all models for *Y*_1_ as it has been found in previous studies that prior infections are associated with the risk of experiencing an infection in the study period ^5^. The β *_CaT_* estimate was obtained from a multivariable logistic regression adjusted for all baseline characteristics measured at second-line treatment initiation, as listed in Table 5. The β *_DiD_* estimate was obtained using logistic difference-in-difference regression and also adjusted for the baseline characteristics at initiation of first- and second-line treatment. Standard IV, CF and the prior outcome augmented IV approaches were fitted to the data using the methods previously described, with adjustment for the same set of baseline covariates in the first and second stage models.

### 5.1 Construction of the Instrumental Variable

As prescription prevalence of both drug classes increased dramatically after 2015 and regional differences in prescribing patterns in the UK exist ^43, 52^, the IV *Z* constructed for this analysis aims to convey information about providers’ preference to prescribe SGTL2i over DPP4i. Preference-based IVs have been proposed when it is assumed that providers prescription preference varies or a substantial variation in practice pattern can be observed ^10, 53^. Hence, for the estimation of β *_IV_, *β *_CF_,* β *_POA–IV_* and β *_POA–CF_,* we constructed a binary IV for patients treated by each respective provider as proposed by Brookhart *et al.* ^10^. As the prescription preference is unobserved, a proxy variable is constructed using the observed prescription behaviour of each provider. Further information about this proxy design can be found for example in Davies *et al.* ^54^ or Widding *et al.* ^55^. The healthcare provider is assumed to have a preference to prescribe SGLT2i over DPP4i depending on the most recent prescription at each point in time. The patient data of the first patient treated within each provider was excluded from the analysis as the IV could not be calculated for this patient. As SGLT2i and DPP4i are newer drug classes and started to be prescribed as second-line treatment more often after 2014 ^43, 44^, we allowed for an initial period in which preference could develop. Therefore, data of individuals initiating second-line treatment from 2016 onwards is analysed. Furthermore, we use an IV which makes it possible to account for changes in prescription preferences ^10^. A similar approach of using clinical commissioning group prescribing history as preference-based IV has been proposed to evaluate T2D treatment by Bidulka *et al.* ^56^.

### 5.2 Results

**Figure 5.**
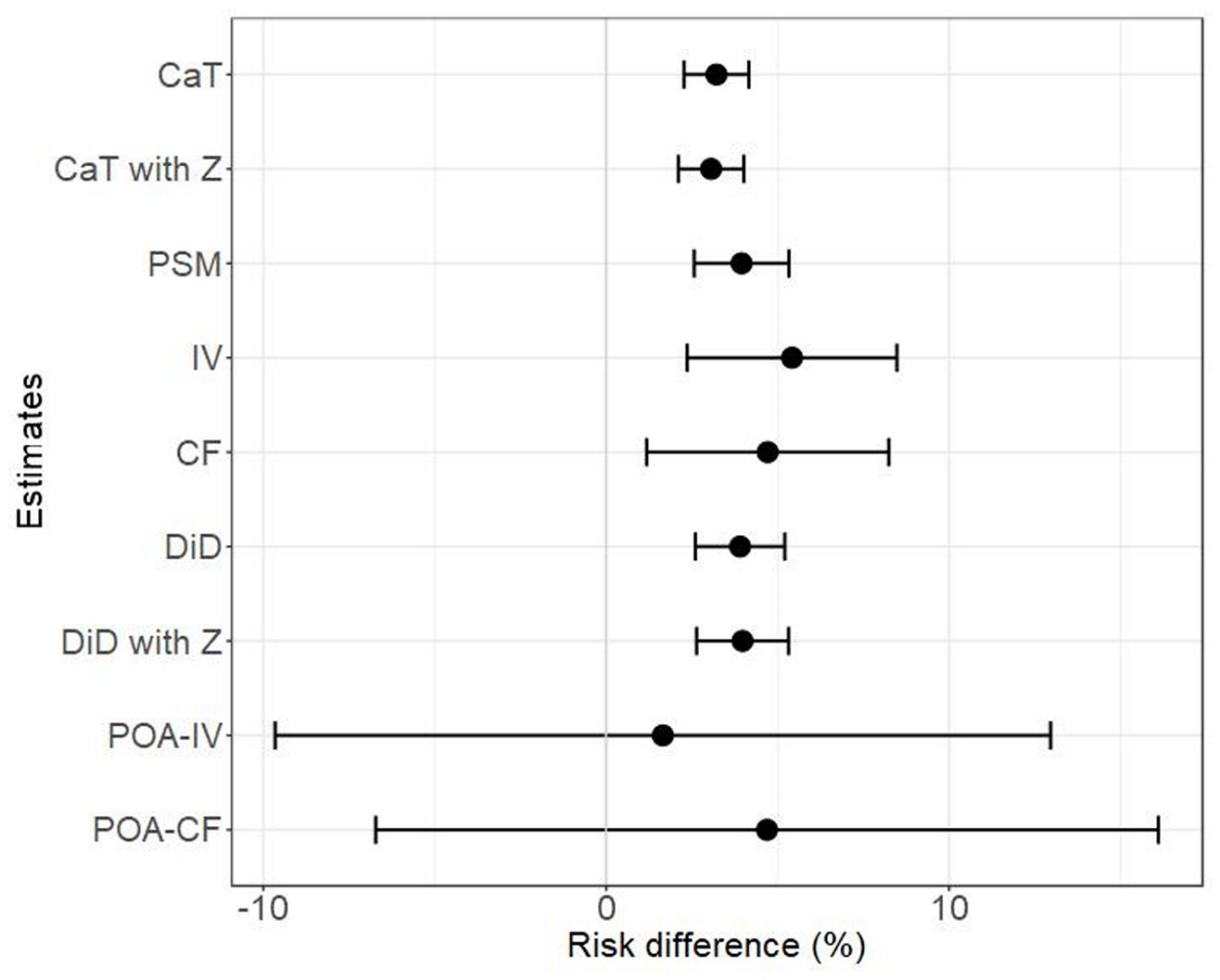
Estimated treatment effect for all estimates and their 95% confidence intervals.

The results of the causal analysis are given in Table 6 and Figure 5. All methods estimate a positive causal effect suggesting that genital infection risk is higher if all people initiated SGLT2i compared to DPP4i. The POA-IV and POA-CF causal estimates are not significantly different from zero at or below the 5% significance threshold. The POA-IV and POA-CF estimate the causal effect with large uncertainty compared to all other approaches and consequently its 95% confidence interval crosses the null. Although the POA-IV and POA-CF estimate can deal with a direct effect of the prior outcome *Y*_0_ on future treatment *X*, it assumes no unmeasured confounding between *Y*_0_ and *X*. Including *Z* in the DiD and CaT model does not result in a big change of the estimation results in this application study.

**Table 6.** Estimation results on risk difference scale (in %), standard error, and p-value of the estimated treatment effect.

Method	Estimate	95% CI	S.E.	p-value
CaT	3.22	2.27; 4.16	0.49	3 *.*10 × 10 ^−14^
CaT with Z	3.06	2.10; 4.01	0.49	2 *.*08 × 10 ^−12^
PSM	3.95	2.57; 5.33	0.72	5 *.*72 × 10 ^−10^
IV	5.42	2.36; 8.48	2.42	0.0003
CF	4.71	1.18; 8.24	2.77	0.008
DiD	3.91	2.60; 5.21	0.66	7 *.*46 × 10 ^−10^
DiD with Z	3.98	2.64; 5.32	0.67	1 *.*16 × 10 ^−9^
POA-IV	1.65	-9.65; 12.96	6.81	0.77
POA-CF	4.69	-6.72; 16.11	6.79	0.42

Table 7 summarized the strength of the IVs measured with the F-statistic of the coefficient of each IV from the first stage regressions of each respective method ^15^. IV and CF as well as POA-IV and POA-CF use the same first stage regression model and the results of the IV strength are therefore summarized in the same row. The instrument strength of *Z* for the IV and CF approach is strong with F-statistic values greater than 10 but *Y*_0_ *Z* is a very weak instrument for the POA-IV and POA-CF approach. This helps to understand the poor results of the two methods which estimate the treatment effect with much higher uncertainty than all other methods applied in this study. Furthermore, it is plausible that *Y*_0_ is confounded by *U* which will also lead to biased results for the POA-IV and POA-CF.

**Table 7.** Strength of the Instrumental Variables measured with the F-statistic of *Z* (for IV and CF) and *Y*_0_ *Z* (POA-IV and POA-CF) from the corresponding first stage regression models.

Models	Instrument	F-statistic
IV and CF	*Z*	345.42
POA-IV and POA-CF	*Y*_0_ *Z*	0.61

### 5.3 Results of the similarity statistic

We now apply our *Q _e_* statistic analysis to the set of estimators to assess their similarity. Table 8 shows the *Q _e_* statistic for a selection of estimator sets. The pairwise correlation of all estimates calculated over 500 bootstrap samples is summarized in Figure 10 in Appendix 7 (Extended data). Interestingly, the test statistic for the closely related CaT and PSM estimates as well as for the POA-IV and POA-CF estimates reveal they are not sufficiently similar even though their values are very close. This is explained by their very high correlation, which *Q _e_* adjusts for. IV and CF are identified as sufficiently similar even after accounting for their correlation. All other selected combinations which do not include CaT or PSM and POA-IV or POA-CF together show that the estimates are statistically similar.

**Table 8.** Test results of the heterogeneity test with *Q _e_* statistic and 95% confidence.

*e*	*Q _e_* statistic	*χ* ^2^ value (df)	p-value	Test decision
CaT, PSM	9.721	3.841 (1)	0.002	not similar
IV, CF	0.093	3.841 (1)	0.761	similar
POA-IV, POA-CF	9.086	3.841 (1)	0.003	not similar
CaT, CF, DiD, POA-CF	2.848	7.815 (3)	0.416	similar
PSM, CF, DiD, POA-CF	0.343	7.815 (3)	0.952	similar
CaT, IV, DiD, POA-IV	4.101	7.815 (3)	0.251	similar
PSM, IV, DiD, POA-IV	1.367	7.815 (3)	0.713	similar

## 6 Summary and conclusion

In this paper we propose a framework for the application of several causal inference methods to assess the comparative effectiveness of two treatments in observational data. This included ‘standard’ confounder adjustment approaches such as multivariable regression and propensity score matching, difference-in-differences and IV estimation. The assumptions of each approach were described, and a simulation was used to assess the impact of violating necessary assumptions on the estimators’ performance. Building on the work of Bowden *et al.* ^35^, we proposed the use of a similarity statistic to formally assess the level of agreement between sets of estimates that can account for their underlying correlation. We hope this statistic could be a useful tool when attempting to triangulate findings from a set of distinct causal estimation strategies going forward.

We illustrated the application of these methods using routinely collected data on people with T2D, to assess the relative safety of SGLT2i compared to DPP4i as second-line therapies on the risk of genital infection. Our heterogeneity analysis showed good agreement between all causal estimates except the PSM/ CaT and POA-IV/POA-CF approaches. In future work, we plan to apply the same causal framework to model alternative T2D outcomes such as HbA1c and other clinically important adverse events. We also plan to extend the approach to fit alternative models that allow for causal effect heterogeneity, so that they may be used in personalised medicine ^57^. Furthermore, the applied analysis showcased how triangulating estimation results with different methods can help to identify implausible results and give further insight in possible assumption violations. Additional work still needs to be done about which estimation results final reports should focus on. Possible strategies could be to only focus on similar estimates or to combine the results.

We proposed the use of the POA-IV/ POA-CF method which is able to leverage an interaction between the prior period and the IV accounting for a possible direct effect of the IV on the outcome and a direct effect of previous outcome events on the treatment decision. Our simulations show that this approach is robust and leads to reliable results in scenarios in which key assumptions of the DiD and the IV approaches are violated, as long as the prior outcome-future treatment relationship does not suffer from unmeasured confounding. Furthermore, our simulation in Section 4.1 showed that POA-IV and POA-CF were less biased than CaT, DiD, IV and CF even if *Y*_0_ was confounded by *U*. As future work we hope to better understand when this will be the case.

As further research, we plan to develop a rigorous hierarchical testing procedure for performing a similarity analysis across an arbitrary number of estimates, whilst controlling the overall family wise error rate. Another approach for combining IV and DiD approaches has recently been proposed by Ye *et al.* ^58^. The ‘instrumented DiD’ purports to offer robustness to time-varying unmeasured confounding and therefore offers considerable utility as an additional estimator within a triangulation analysis.

## 7.4 Ethics and consent statement

This article is based in part on data from the Clinical Practice Research Datalink obtained under licence from the UK Medicines and Healthcare products Regulatory Agency. CPRD data is provided by patients and collected by the NHS as part of their care and support. Approval for CPRD data access and the study protocol was granted by the CPRD Independent Scientific Advisory Committee (eRAP protocol number: 22 002000, approved on 19/09/2022). Informed consent was waived for this retrospective study as CPRD patient data is anonymized. Individual patients can opt-out of sharing their data for CPRD, and CPRD does not collect data for these patients. All methods were carried out in accordance with relevant guidelines and regulations. The CPRD adheres to all UK and European laws and guidelines governing research. All data provided by the CPRD is anonymized.

## Data Availability

Data from the Clinical Practice Research Datalink (CPRD) used in this study are accessible only upon application due to privacy and data sharing restrictions. Access to CPRD data is governed by the CPRD Independent Scientific Advisory Committee (ISAC), which reviews applications to ensure data protection and ethical use. Researchers interested in accessing CPRD data can apply via the CPRD's data access page, available at CPRD Data Access. Access requests must meet specific conditions, including ethical approval and a clear justification of the research purpose. Data use is restricted to approved research projects, and applicants are required to demonstrate adherence to CPRD's security and governance protocols. Due to the sensitive nature of patient information, CPRD data cannot be openly shared, and individual patient consent is not feasible as data are anonymized for research purposes. Further information on application procedures, requirements, and ISAC guidelines can be found on the CPRD website. Researchers and reviewers must apply directly to CPRD and meet the necessary criteria to access these data. Applicants must register on the submission system, supply curriculum vitae information, and complete a research protocol, which is then reviewed by an independent committee. Zenodo: Appendix - Triangulating Instrumental Variable, confounder adjustment and difference-in-difference methods for comparative effectiveness research in observational data. https://doi.org/10.5281/zenodo.14055585 ^59^. **The project contains following extended data:** Guedemann_etal_2024_Appendix.pdf Data are available under the terms of the Creative Commons Attribution 4.0 International license (CC-BY 4.0) Zenodo: STROBE checklist for ‘Triangulating Instrumental Variable, confounder adjustment and difference-in-difference methods for comparative effectiveness research in observational data’. https://zenodo.org/records/14204680 ^14^. Data are available under the terms of the Creative Commons Attribution 4.0 International license (CC-BY 4.0)

## Software availability statement

**Software available from:** The software used in this study includes R and RStudio.

**Source code available from:** https://github.com/GuedemannLaura/POA-IV

**Archived software available from:** https://doi.org/10.5281/zenodo.14136128 ^13^.

**License:** The software used in this study is available under the MIT License, an OSI-approved open-source license.

## References

[1] Greenland S Morgenstern H : Confounding in health research. *Annu Rev Public Health.* 2001;22:189–212. 10.1146/annurev.publhealth.22.1.189 11274518

[2] Jager KJ Zoccali C MacLeod A : Confounding: what it is and how to deal with it. *Kidney Int.* Nature Publishing Group,2008;73(3):256–260. 10.1038/sj.ki.5002650 17978811

[3] Boyko EJ : Observational research - opportunities and limitations. *J Diabetes Complications.* 2013;27(6):642–648. 10.1016/j.jdiacomp.2013.07.007 24055326 PMC3818421

[4] Dawwas GK Smith SM Park H : Cardiovascular outcomes of sodium glucose cotransporter‐2 inhibitors in patients with type 2 diabetes. *Diabetes Obes Metab.* 2019;21(1):28–36. 10.1111/dom.13477 30039524

[5] McGovern AP Hogg M Shields BM : Risk factors for genital infections in people initiating SGLT2 inhibitors and their impact on discontinuation. *BMJ Open Diabetes Res Care.* BMJ Publishing Group,2020;8(1): e001238. 10.1136/bmjdrc-2020-001238 32448787 PMC7252998

[6] Streeter AJ Lin NX Crathorne L : Adjusting for unmeasured confounding in nonrandomized longitudinal studies: a methodological review. *J Clin Epidemiol.* 2017;87:23–34. 10.1016/j.jclinepi.2017.04.022 28460857 PMC5589113

[7] Bowden J Bornkamp B Glimm E : Connecting instrumental variable methods for causal inference to the estimand framework. *Stat Med.* in press,2021;40(25):5605–5627. 10.1002/sim.9143 34288021

[8] Pezzin LE Laud P Yen TWF : Re-examining the relationship of breast cancer hospital and surgical volume to mortality: an instrumental variable analysis. *Med Care.* NIH Public Access,2015;53(12):1033–9. 10.1097/MLR.0000000000000439 26492213 PMC4648647

[9] Smith GD Ebrahim S : ‘Mendelian randomization’: can genetic epidemiology contribute to understanding environmental determinants of disease? *Int J Epidemiol.* 2003;32(1):1–22. 10.1093/ije/dyg070 12689998

[10] Brookhart MA Wang PS Solomon DH : Evaluating short-term drug effects using a physician-specific prescribing preference as an instrumental variable. *Epidemiology.* 2006;17(3):268–275. 10.1097/01.ede.0000193606.58671.c5 16617275 PMC2715942

[11] Rodgers LR Dennis JM Shields BM : Prior event rate ratio adjustment produced estimates consistent with randomized trial: a diabetes case study. *J Clin Epidemiol.* Elsevier USA,2020;122:78–86. 10.1016/j.jclinepi.2020.03.007 32194148 PMC7262589

[12] Lin NX Henley WE : Prior event rate ratio adjustment for hidden confounding in observational studies of treatment effectiveness: a pairwise Cox likelihood approach. *Stat Med.* John Wiley and Sons Ltd,2016;35(28):5149–5169. 10.1002/sim.7051 27477530 PMC5111612

[13] Güdemann LM Dennis JM McGovern AP : Appendix - Triangulating Instrumental Variable, confounder adjustment and difference-in-difference methods for comparative effectiveness research in observational data. 2024. 10.5281/zenodo.14136128 PMC12504944 41069648

[14] Güdemann LM Dennis JM McGovern AP : Appendix - Triangulating Instrumental Variable, confounder adjustment and difference-in-difference methods for comparative effectiveness research in observational data. 2024. 10.5281/zenodo.14204680 PMC12504944 41069648

[15] Stock JH Wright JH Yogo M : A survey of weak instruments and weak identification in generalized method of moments. *J Bus Econ Stat.* 2002;20(4):518–529. 10.1198/073500102288618658

[16] Aso S Yasunaga H : Introduction to instrumental variable analysis. 2020;2(3):69–74. 10.37737/ace.2.3_69

[17] Lousdal ML : An introduction to instrumental variable assumptions, validation and estimation. *Emerg Themes Epidemiol.* BioMed Central Ltd,2018;15:1–7. 10.1186/s12982-018-0069-7 29387137 PMC5776781

[18] Wooldridge JM : Introductory Econometrics - A Modern Approach.7th ed., South-Western College Publishing,2019.

[19] Bowden RJ Turkington DA : Instrumental Variables.Cambridge: Cambridge University Press,1984.

[20] Weiner MG Xie D Tannen RL : Replication of the Scandinavian Simvastatin Survival study using a primary care medical record database prompted exploration of a new method to address unmeasured confounding. *Pharmacoepidemiol Drug Saf.* Wiley Online Library,2008;17(7):661–670. 10.1002/pds.1585 18327857

[21] Tannen RL Weiner MG Xie D : Replicated studies of two randomized trials of angiotensin‐converting enzyme inhibitors: further empiric validation of the ‘prior event rate ratio’ to adjust for unmeasured confounding by indication. *Pharmacoepidemiol Drug Saf.* Wiley Online Library,2008;17(7):671–685. 10.1002/pds.1584 18327852

[22] Uddin MJ Groenwold RHH van Staa TP : Performance of prior event rate ratio adjustment method in pharmacoepidemiology: a simulation study. *Pharmacoepidemiol Drug Saf.* Wiley Online Library,2015;24(5):468–477. 10.1002/pds.3724 25410590

[23] Gallagher AM de Vries F van Staa T : Prior event rate ratio adjustment: a magic bullet or more of the same? *Pharmacoepidemiol Drug Saf.* JOHN WILEY & SONS LTD THE ATRIUM, SOUTHERN GATE, CHICHESTER PO19 8SQ, W …,2009;18:S14–S15. Reference Source

[24] Yu M Xie D Wang X : Prior event rate ratio adjustment: numerical studies of a statistical method to address unrecognized confounding in observational studies. *Pharmacoepidemiol Drug Saf.* Wiley Online Library,2012;21(Suppl 2):60–68. 10.1002/pds.3235 22552981

[25] Zhou H Taber C Arcona S : Difference-in-differences method in comparative effectiveness research: utility with unbalanced groups. *Appl Health Econ Health Policy.* Springer,2016;14(4):419–429. 10.1007/s40258-016-0249-y 27371369 PMC4937082

[26] Lopez Bernal J Cummins S Gasparrini A : Difference in difference, controlled interrupted time series and synthetic controls. *Int J Epidemiol.* Oxford University Press,2019;48(6):2062–2063. 10.1093/ije/dyz050 30904926

[27] Craig P Cooper C Gunnell D : Using natural experiments to evaluate population health interventions: new Medical Research Council guidance. *J Epidemiol Community Health.* BMJ Publishing Group Ltd,2012;66(12):1182–1186. 10.1136/jech-2011-200375 22577181 PMC3796763

[28] Rockers PC Røttingen JA Shemilt I : Inclusion of quasi-experimental studies in systematic reviews of health systems research. *Health Policy.* Elsevier,2015;119(4):511–521. 10.1016/j.healthpol.2014.10.006 25776033

[29] Soumerai SB Starr D Majumdar SR : How do you know which health care effectiveness research you can trust? A guide to study design for the perplexed. *Prev Chronic Dis.* 2015;12: E101. 10.5888/pcd12.150187 26111157 PMC4492215

[30] Tchetgen ET : A note on the control function approach with an instrumental variable and a binary outcome. *Epidemiol Methods.* De Gruyter,2014;3(1):107–112. 10.1515/em-2014-0009 25914867 PMC4405241

[31] Leeper TJ : margins: marginal effects for model objects, r package version 0.3.26. 2021. Reference Source

[32] Huitfeldt A Stensrud MJ Suzuki E : On the collapsibility of measures of effect in the counterfactual causal framework. *Emerg Themes Epidemiol.* 2019;16:1–5. 10.1186/s12982-018-0083-9 30627207 PMC6322247

[33] Slotani M : Tolerance regions for a multivariate normal population. *Ann Inst Stat Math.* Springer,1964;16:135–153. 10.1007/BF02868568

[34] Cochran WG : The combination of estimates from different experiments. *Biometrics.* JSTOR,1954;10(1):101–129. 10.2307/3001666

[35] Bowden J Pilling LC Türkmen D : The Triangulation WIthin a STudy (TWIST) framework for causal inference within pharmacogenetic research. *PLoS Genet.* Public Library of Science San Francisco, CA USA,2021;17(9): e1009783. 10.1371/journal.pgen.1009783 34495953 PMC8452063

[36] R Core Team: R: A language and environment for statistical computing. R foundation for statistical computing.Vienna, Austria,2023. Reference Source

[37] Posit team: RStudio: integrated development environment for R. Posit Software.PBC, Boston, MA,2023. Reference Source

[38] Morris TP White IR Crowther MJ : Using simulation studies to evaluate statistical methods. *Stat Med.* John Wiley and Sons Ltd,2019;38(11):2074–2102. 10.1002/sim.8086 30652356 PMC6492164

[39] Small DS : Mediation analysis without sequential ignorability: using baseline covariates interacted with random assignment as instrumental variables. 2012;46:91–103. 10.48550/arXiv.1109.1070 PMC4244702 25435642

[40] Spiller W Slichter W Bowden J : Detecting and correcting for bias in Mendelian randomization analyses using gene-by-environment interactions. *Int J Epidemiol.* 2019;48(3):702–712. 10.1093/ije/dyy204 30462199 PMC6659360

[41] National Institute for Health and Care Excellence: Type 2 diabetes in adults: management. 2020. Reference Source

[42] Buse JB Wexler DJ Tsapas A : 2019 Update to: Management of Hyperglycemia in Type 2 Diabetes, 2018. A consensus Report by the American Diabetes Association (ADA) and the European Association for the Study of Diabetes (EASD). *Diabetes Care.* 2020;43(2):487–493. 10.2337/dci19-0066 31857443 PMC6971782

[43] Dennis JM Henley WE McGovern AP : Time trends in prescribing of type 2 diabetes drugs, glycaemic response and risk factors: a retrospective analysis of primary care data. *Diabetes Obes Metab.* 2019;21(7):1576–1584. 10.1111/dom.13687 30828962 PMC6618851

[44] Montvida O Shaw J Atherton JJ : Long-term trends in Antidiabetes drug usage in the US: real-world evidence in patients newly diagnosed with Type 2 diabetes. *Diabetes Care.* 2018;41(1):69–78. 10.2337/dc17-1414 29109299

[45] Dennis JM : Precision medicine in type 2 diabetes: using individualized prediction models to optimize selection of treatment. *Diabetes.* 2020;69(10):2075–2085. 10.2337/dbi20-0002 32843566 PMC7506836

[46] Herrett E Gallagher AM Bhaskaran K : Data resource profile: Clinical Practice Research Datalink (CPRD). *Int J Epidemiol.* 2015;44(3):827–36. 10.1093/ije/dyv098 26050254 PMC4521131

[47] Rodgers LR Weedon MN Henley WE : Cohort profile for the MASTERMIND study: using the Clinical Practice Research Datalink (CPRD) to investigate stratification of response to treatment in patients with type 2 diabetes. *BMJ Open.* 2017;7(10): e017989. 10.1136/bmjopen-2017-017989 29025846 PMC5652624

[48] Booth CM Tannock IF : Randomised controlled trials and population-based observational research: partners in the evolution of medical evidence. *Br J Cancer.* 2014;110(3):551–5. 10.1038/bjc.2013.725 24495873 PMC3915111

[49] Hinton W Feher M Munro N : Real-world prevalence of the inclusion criteria for the LEADER trial: data from a national general practice network. *Diabetes Obes Metab.* 2019;21(7):1661–1667. 10.1111/dom.13710 30900349 PMC6619442

[50] Vandenbroucke JP Elm Ev Altman DG : Strengthening the Reporting of Observational Studies in Epidemiology (STROBE): explanation and elaboration. *Ann Intern Med.* 2007;147(8):W163–94. 10.7326/0003-4819-147-8-200710160-00010-w1 17938389

[51] Ho DE Imai K King G : {MatchIt}: nonparametric preprocessing for parametric causal inference. *J Stat Softw.* 2011;42(8):1–28. 10.18637/jss.v042.i08

[52] Curtis HJ Dennis JM Shields BM : Time trends and geographical variation in prescribing of drugs for diabetes in England from 1998 to 2017. *Diabetes Obes Metab.* 2018;20(9):2159–2168. 10.1111/dom.13346 29732725 PMC6099452

[53] Korn EL Baumrind S : Clinician preferences and the estimation of causal treatment differences. *Statist Sci.* 1998;13(3):209–235. 10.1214/ss/1028905885

[54] Davies NM Thomas KH Taylor AE : How to compare instrumental variable and conventional regression analyses using negative controls and bias plots. *Int J Epidemiol.* 2017;46(6):2067–2077. 10.1093/ije/dyx014 28398582 PMC5837536

[55] Widding-Havneraas T Chaulagain A Lyhmann I : Preference-based instrumental variables in health research rely on important and underreported assumptions: a systematic review. *J Clin Epidemiol.* 2021;139:269–278. 10.1016/j.jclinepi.2021.06.006 34126207

[56] Bidulka P O'Neill S Basu A : Protocol for an observational cohort study investigating personalised medicine for intensification of treatment in people with type 2 diabetes mellitus: the PERMIT study. *BMJ Open.* 2021;11(9): e046912. 10.1136/bmjopen-2020-046912 34580091 PMC8477338

[57] Dennis JM Young KG McGovern AP : Derivation and validation of a type 2 diabetes treatment selection algorithm for SGLT2-inhibitor and DPP4-inhibitor therapies based on glucose-lowering efficacy: cohort study using trial and routine clinical data. *medRxiv.* 2021. 10.1101/2021.11.11.21265959

[58] Ye T Ertefaie A Flory J : Instrumented Difference-in-Differences. *arXiv: 2011.03593 [stat.ME].* 2020. 10.48550/arXiv.2011.03593

[59] Güdemann LM Dennis JM McGovern AP : Appendix - Triangulating Instrumental Variable, confounder adjustment and difference-in-difference methods for comparative effectiveness research in observational data. *Zenodo.* 2024. 10.5281/zenodo.14055585 PMC12504944 41069648

