# Supplementary material for: Triangulating Instrumental Variable, confounder adjustment and difference-in-difference methods for comparative effectiveness research in observational data
Source: Wellcome Open Res. 2025 Aug 19;10:54. Originally published 2025 Feb 10. [Version 2] doi: 10.12688/wellcomeopenres.22955.2 (PMC12504944; doi:10.12688/wellcomeopenres.22955.2)
Supplement: Peer review history

# Reviewer report: Reviewer response for version 2

**DOI:** 10.21956/wellcomeopenres.27285.r131348

**Published:** 2025-10-07

**Authors:** Paul Zivich

**Affiliations:** 1 University of North Carolina at Chapel Hill, Chapel Hill, North Carolina, USA

The authors review several common strategies for estimation of the average treatment effect. Given these different strategies and their reliance on differing sets of assumptions, the authors consider how we might be able to triangulate these results into a more reliable estimate. Finally, the authors propose a modified IV method and a test for the similarity of estimates. My comments are organized in the following way: first I explain my rationale for selection of 'Partly' to the prior questions and then I state some general recommendations.

 Below is my rationale for selecting 'Partly' for the study design being appropriate and technically sound:

 - I mostly agree with Reviewer 1, but I would note that the gold-standard should be a RCT with no censoring with participants from a random sample of the target population. This later part is important as it defines the context for which our estimate applies. Both internal and external validity are important, with most trial designs focusing on only internal validity (hence not a true gold-standard).

 - Equation 2 is only correct in the case where Y is a continuous outcome modeled using linear regression without interaction terms. It is not generally the case that E[E[Y | X]] = E[Y | X]. In this more general case, \beta_{CaT} would be defined as E[E[Y_1 | X=x, W_1]].

 - I would have liked to see a bit more formal derivations of POA-IV and POA-CF. Right now the authors provide some plain language descriptions of the assumptions. I personally would have liked to see some sort of formal identification proof with the assumptions expressed mathematically (in addition to the plain language summaries). An example of what I have in mind can be found in Ref 1. I also think this approach might be related to Bespoke Instrumental variables, but I am not entirely sure if this is the case. See Ref 3 for more on Bespoke Instruments.

 - I do not find the authors' response to Reviewer 1's Comment 20 (regarding the use of propensity score matching) to be sufficient. I share the same concern as Reviewer 1, as propensity score matching changes the parameter by changing the target population. So the propensity score matching results in Table 6 and Figure 5 are not directly comparable to other methods, like if we instead used IV to estimate the local average treatment effect. Given that 1/3 of the data was discarded during matching, this is likely to noticeably change the underlying population distribution of W_1. I recognize prior literature has elided this issue, but that does not mean we should repeat their mistakes in the name of comparability.

 - I have a concern regarding the applied example. In the applied example, the authors appear to have time-to-event data, not outcomes that are measured at a single time for all units. Given this data structure, the estimation methods in Sections 2 and 4 are not directly applicable, as these expression are not identified with right censored data under the provided assumptions. I recognize that this application may be mainly to demonstrate the proposed method, but I do not think the methods are fully appropriate for the selected example.

 Below is my rationale for selecting 'Partly' for reproducibility:

 - I appreciate the authors organization of their GitHub code and use of Zenodo. My recommendation is to add the versions of the packages into the README file or into R_packages.R. Without this version control, results may not be reproducible into the future.

 The following are my additional comments and suggestions:

 - One extension the authors may want to comment on is estimators that are robust to differing assumptions. I think the ideal development would be as estimator that combines the different methods together in such a way that the estimator would be unbiased as long as one of the methods is unbiased. A similar concept occurs with so-called doubly-robust estimators, which combine outcome models and propensity scores in such a way that the estimator is unbiased as long as one of the two models is specified. See Ref 2. To me, this seems like the ideal methodological development in triangulation methods.

 - As an alternative way of framing their test, the authors might consider drawing inspiration of equivalence tests in the historic controls literature. See Ref 4.

Is the work clearly and accurately presented and does it cite the current literature?

Yes

If applicable, is the statistical analysis and its interpretation appropriate?

Yes

Are all the source data underlying the results available to ensure full reproducibility?

Partly

Is the study design appropriate and is the work technically sound?

Partly

Are the conclusions drawn adequately supported by the results?

Yes

Are sufficient details of methods and analysis provided to allow replication by others?

Yes

Reviewer Expertise:

Epidemiologic methods, including propensity score methods.

I confirm that I have read this submission and believe that I have an appropriate level of expertise to confirm that it is of an acceptable scientific standard, however I have significant reservations, as outlined above.

## References

[1] : An Introduction to Proximal Causal Inference. *Statistical Science* .2024;39(3) : 10.1214/23-STS911 10.1214/23-STS911

[2] : Doubly Robust Estimation of Causal Effects. *American Journal of Epidemiology* .2011;173(7) : 10.1093/aje/kwq439 761-767 10.1093/aje/kwq439 21385832 PMC3070495

[3] : Bespoke Instruments: A new tool for addressing unmeasured confounders. *American Journal of Epidemiology* .2022;191(5) : 10.1093/aje/kwab288 939-947 10.1093/aje/kwab288 PMC943046834907434

[4] : Revisit of test‐then‐pool methods and some practical considerations. *Pharmaceutical Statistics* .2020;19(5) : 10.1002/pst.2009 498-517 10.1002/pst.2009 32171048

# Reviewer report: Reviewer response for version 2

**DOI:** 10.21956/wellcomeopenres.27285.r129959

**Published:** 2025-09-10

**Authors:** Zach Shahn

**Affiliations:** 1 CUNY School of Public Health, New York, USA

I appreciate the authors' responsiveness to the initial round of reviews. There are a couple of points where I wasn't appeased by the response, but I'll mention them here and not officially recommend further revisions but leave it to the authors.

 1) The methods generally target different estimands. The confounding adjustment method targets the ATE, DiD targets the ATT, and IV targets the ATT or the CACE depending on which 'fourth assumption' is made. Only under additional effect homogeneity assumptions which are not discussed would these three methods be expected to estimate the same quantity, even if they were all unbiased. Perhaps I buried this comment too deeply in the first review.

 2) In the response to reviews, the authors justify omitting arrows from their DAGs by saying that they want to focus on core assumptions. However, arrow omissions represent additional assumptions. While sparser graphs might be less visually cluttered, they are much more conceptually cluttered, which is what resulted in both Reviewer 1 and myself being confused about why certain arrows are missing.

 3) The interaction that is used as an IV really only is an IV within a specific linear model and not generally. I encourage the authors to revisit this issue

Is the work clearly and accurately presented and does it cite the current literature?

Yes

If applicable, is the statistical analysis and its interpretation appropriate?

Partly

Are all the source data underlying the results available to ensure full reproducibility?

Yes

Is the study design appropriate and is the work technically sound?

Partly

Are the conclusions drawn adequately supported by the results?

Partly

Are sufficient details of methods and analysis provided to allow replication by others?

Yes

Reviewer Expertise:

Causal inference, statistics

I confirm that I have read this submission and believe that I have an appropriate level of expertise to confirm that it is of an acceptable scientific standard.

# Reviewer report: Reviewer response for version 1

**DOI:** 10.21956/wellcomeopenres.25276.r120595

**Published:** 2025-04-09

**Authors:** Zach Shahn

**Affiliations:** 1 CUNY School of Public Health, New York, USA

First, I echo literally all of Reviewer 1’s comments, including the complimentary pre-amble. Thank you, authors, for writing an interesting paper, and thank you, Reviewer 1, for making my job much easier by going first. (Even though this isn't anonymous, it still somehow feels strange to shed the usual review conventions and use names!)  I tried to avoid repeating points from the first review, but there may be some overlap. I have some additional persnickety comments about language, as well as some comments about substance and framing.

 Regarding framing, I find the application quite convincing, but it’s because of Figure 5 and the simulations in Section 3 rather than the similarity statistic, which I think should be de-emphasized. (It seems like any statistical statements you want to make about the differences between the statistical estimands targeted by the three estimators would be best summed up by confidence intervals for summary statistics of interest derived from their joint bootstrap distribution. I don’t see the benefit of putting this into a hypothesis testing framework, even equivalence testing.) The simulations seem to indicate that violations of various assumptions do not tend to bias the different estimators in similar ways, which makes their agreement more convincing. This logic should perhaps be more explicitly emphasized. The most reasonable explanation for the results appears to be that here is very little unobserved confounding (or confounding by Y0) relative to the effect size, and the exclusion restriction approximately holds. (Is this the only scenario where triangulation via agreement of DiD, IV, and confounding adjustment methods would yield a conclusive positive result?)

 A general question I have is how to choose which estimators to toss into the triangulation pot. For example, proximal inference would be a natural extra method to try, but maybe there is no need because we’re already convinced, and if its assumptions failed it would only muddy the waters. Is it then risky to perform the analysis? (It feels counterintuitive that performing extra analyses might be somehow bad, but within this framework it appears to possibly be?) How to decide then which methods to include, in what order (if it’s a sequential process), and what a valid stopping rule should be? This question is likely beyond the scope of the paper. Even if so, a comment remains that it was unclear which aspects of the triangulation process illustrated in the paper are meant to be generalizable to other settings. It would be illuminating to include some discussion of why and how these methods in particular were chosen for triangulation in this setting, even if it was somewhat ad hoc.

**Below are comments about specific snippets from the paper:**

**Section 1**

 > *“* *This opens up the possibility that treatment choice (or the extent of treatment received) and patient outcomes may be simultaneously predicted by common variables, which could bias the analysis because of a lack of balance across treatment groups, in contrast to the adherence affected RCT setting previously discussed.”*

 The language “simultaneously predicted” is imprecise here. It could apply to M-bias type scenarios where there would not actually be confounding.

*>“The IV approach addresses confounding by substituting each patients’ observed treatment with a predicted treatment. This prediction is made using a variable (the instrument) that is assumed to be independent of any confounders and only affects the outcome through the treatment.”*

 This describes the mechanics of two stage least squares, which is just one of many estimators consistent for causal effects under IV assumptions.

*>“Randomization to a treatment group within a RCT is perhaps the best example of an IV”*

 Assuming randomization only impacts the outcome via received treatment, which is not always true.

*>“Examples of IVs for observational data include…”*

 These are variables that past authors have asserted are IVs, but not necessarily valid IVs.

 >The PERR approach seems also to have deep connections to Sofer et al (2016).

 >In Figure 1, W0 has no direct effect on Y1 not through W1, and both W0 and W1 are completely independent of U. None of these things seem necessary for any of the methods to work. Also, Reviewer 1 noted that there is no Y0-->Y1 arrow and asked whether this is necessary for any results. I would note that for DiD the presence of such an arrow does indeed make the identifying assumptions less plausible because the direct association between U and Y0 through the U-->Y0 arrow needs to coincidentally equal the total association between U and Y1 both through the direct U-->Y1 arrow and the indirect U-->Y0-->Y1 path. This is discussed in Kim and Steiner (2021)

 >“Complete adherence could be illustrated in Figure 1 by letting *Z* represent the randomized treatment assignment and removing all arrows into *X* from *W*_0_, *W*_1_ and *U*, so that only the *Z* → *X* arrow remains.”

 Yes, but this wouldn’t fully capture it. You’d also need the Z-->X relationship to be deterministic.

**Section 2**

 >The RHS of Equation (2) depends on W1 and is not the ATE except under strong effect homogeneity assumptions like in the linear model introduced after equation (2).

 >“This could be estimated from fitting the following multivariable regression of Y _1_ on X and W _1_ as…”

 Under extra parametric modeling assumptions.

 >The DiD section should also state the conditional parallel trends assumption (Abadie, 2005) as the assumption under which DiD is traditionally justified. Determining when parallel trends holds in terms of confounding is helpful and has been considered elsewhere. The proof of the sufficiency of DiD1 and DiD2 in Appendix 2 assumes linear models and is not general. Kim and Steiner (2021) should be cited concerning conditions under which parallel trends holds given linear models. Ghanem et al (2022) should be cited concerning more general structural conditions under which parallel trends holds. Sofer et al (2016) discuss how DiD can be justified under additive equi-confounding assumptions similar to your framing.

 >(6) is incorrect. It is missing an outer expectation over the covariates. Abadie (2005) provides singly robust estimators based on standardization or IPW, and Sant’anna and Zhao (2020) provide doubly robust ones.

 >(7) again makes strong parametric linear assumptions that aren’t explicitly stated and are not necessary. Maybe state at some point early on that the paper will consider simple parametric implementations of all methods to focus on the issue of triangulation. (I actually think that using DML with cross-fitting would put more focus on triangulation by removing nuisance model mis-specification as a factor.)

 >Finally, DiD estimates the effect of treatment on the treated (ATT), not the ATE. This should be stated, along with the extra assumption you’d require (as in the IV setting) that the ATT is equal to the ATE.

 >The CF abbreviation is used before it’s introduced.

 >Regarding binary outcomes, if the proportions in the treatment groups aren’t too close to 0 or 1 for parallel trends to make sense, it’s fine to just use a standard DiD estimator. (This is what you appear to have done in your simulations, judging from Table 1?) Otherwise, you might also want to mention the Universal DiD approach of Park and Tchetgen Tchetgen (2022). Also see the summary of other alternatives for DiD with binary outcomes therein. And it would be helpful if you cited the paper and assumptions justifying your suggested approach. Is it Wooldridge (2022) where parallel trends are now assumed on the logit scale?

**Section 3**

 >“the DiD1 assumption which requires *X* to be independent of *Y*_0_"

 It just requires X to not be caused by Y0

**Section 4**

 >“aims to overcome the limitations of the DiD and standard IV”

 The framing of the POA-IV as a weakening of the assumptions required for DiD or IV is misleading. The method requires additional parametric assumptions in the form of the linear models (which should be explicitly stated as assumptions, including the point that Reviewer 1 made about the absence of the interaction in the outcome model being crucial) and also the assumption that U is not a cause of Y0. I would think it’s quite unusual for this latter assumption (that an unobserved confounder impacts the later measurement but not the earlier measurement of an outcome) to hold. Granted, the method seemed to have mild bias due to failure of this assumption in the simulations, but it’s not clear if this would generally be the case. It might be worth mentioning (if correct) that if there *is* unobserved confounding, POA and DiD would almost never both give the right answer since parallel trends requires a U-->Y0 arrow and POA requires its absence. This could potentially make it a particularly good method to include with DiD in a triangulation study? 

 The DAG in Figure 4 including an arrow from Y0Z to X in addition to the arrows from Y0 and Z into X seems to reflect parametric assumptions and not really be a formal causal DAG, which is nonparametric and would not have the Y0Z to X arrow since Y0Z is a deterministic function of Y0 and Z.

**Section 6**

 >“Another approach for combining IV and DiD approaches has recently been proposed by Ye *et al.* ^58^. The ‘instrumented DiD’ purports to offer robustness to time-varying unmeasured confounding and therefore offers considerable utility as an additional estimator within a triangulation analysis.”

 This is true, but they do assume a different data structure with treatments at two time-points. And again, I’d be interested in what are the criteria for determining level of “utility as an additional estimator within a triangulation analysis.”

Is the work clearly and accurately presented and does it cite the current literature?

Yes

If applicable, is the statistical analysis and its interpretation appropriate?

Partly

Are all the source data underlying the results available to ensure full reproducibility?

Yes

Is the study design appropriate and is the work technically sound?

Partly

Are the conclusions drawn adequately supported by the results?

Partly

Are sufficient details of methods and analysis provided to allow replication by others?

Yes

Reviewer Expertise:

Causal inference, statistics

I confirm that I have read this submission and believe that I have an appropriate level of expertise to confirm that it is of an acceptable scientific standard, however I have significant reservations, as outlined above.

## References

[1] : On negative outcome control of unobserved confounding as a generalization of difference-in-differences. *Stat Sci* .2016;31(3) : 10.1214/16-STS558 348-361 10.1214/16-STS558 28239233 PMC5322866

[2] Semiparametric Difference-in-Differences Estimators, Abadie, A. *The Review of Economic Studies* .(2005) 72(1), 1–19; Reference source

[3] : Gain Scores Revisited: A Graphical Models Perspective. *Sociological Methods & Research* .2021;50(3) : 10.1177/0049124119826155 1353-1375 10.1177/0049124119826155

[4] Selection and parallel trends Ghanem, Dalia, Pedro HC Sant'Anna, and Kaspar Wüthrich. *arXiv preprint* .2022); Reference source

[5] : Doubly robust difference-in-differences estimators. *Journal of Econometrics* .2020;219(1) : 10.1016/j.jeconom.2020.06.003 101-122 10.1016/j.jeconom.2020.06.003

[6] : Universal Difference-in-Differences for Causal Inference in Epidemiology. *Epidemiology* .2024;35(1) : 10.1097/EDE.0000000000001676 16-22 10.1097/EDE.0000000000001676 38032801 PMC10683972

[7] A universal difference-in-differences approach for causal inference, Park, Chan, and Eric Tchetgen Tchetgen. *arXiv preprint* .(2022); Reference source 10.1097/EDE.0000000000001676PMC1068397238032801

[8] : Simple approaches to nonlinear difference-in-differences with panel data. *The Econometrics Journal* .2023;26(3) : 10.1093/ectj/utad016 C31-C66 10.1093/ectj/utad016

# Response

**Published:** 2025-07-31

**Authors:** Laura Güdemann

**Affiliations:** Faculty of Health and Life Sciences, University of Exeter, Exeter, England, UK

**We are grateful for the time and effort devoted to reviewing our work and for the supportive comments.** **Comment 1:** Regarding framing, I find the application quite convincing, but it’s because of Figure 5 and the simulations in Section 3 rather than the similarity statistic, which I think should be de-emphasized. (It seems like any statistical statements you want to make about the differences between the statistical estimands targeted by the three estimators would be best summed up by confidence intervals for summary statistics of interest derived from their joint bootstrap distribution. I don’t see the benefit of putting this into a hypothesis testing framework, even equivalence testing.) The simulations seem to indicate that violations of various assumptions do not tend to bias the different estimators in similar ways, which makes their agreement more convincing. This logic should perhaps be more explicitly emphasized. The most reasonable explanation for the results appears to be that here is very little unobserved confounding (or confounding by Y0) relative to the effect size, and the exclusion restriction approximately holds. (Is this the only scenario where triangulation via agreement of DiD, IV, and confounding adjustment methods would yield a conclusive positive result?) **Answer:** We have addressed previous helpful comments from reviewer 1 regarding the proposed dissimilarity statistics and clarified the interpretation of the results. The main aspect of our study remain the two extensively discussed simulation studies with multiple scenarios and the application study. We have extended the discussion of the results in Section 5 (Section 5.2) to emphasize the logic of the triangulation including the results of out simulation studies.

   “The results of the causal analysis are given in Table 6 and Figure 5. All methods estimate a positive causal effect suggesting that genital infection risk is higher if all people initiated SGLT2i compared to DPP4i. As seen in simulation studies outlined in previous sections, the methods utilize different parts of the data and require different assumptions. When triangulating results from these estimation methods, their agreement gives confidence in the detection of an increased risk of genital infections of SGLT2i.” **Comment 2:** A general question I have is how to choose which estimators to toss into the triangulation pot. For example, proximal inference would be a natural extra method to try, but maybe there is no need because we’re already convinced, and if its assumptions failed it would only muddy the waters. Is it then risky to perform the analysis? (It feels counterintuitive that performing extra analyses might be somehow bad, but within this framework it appears to possibly be?) How to decide then which methods to include, in what order (if it’s a sequential process), and what a valid stopping rule should be? This question is likely beyond the scope of the paper. Even if so, a comment remains that it was unclear which aspects of the triangulation process illustrated in the paper are meant to be generalizable to other settings. It would be illuminating to include some discussion of why and how these methods in particular were chosen for triangulation in this setting, even if it was somewhat ad hoc. **Response:** We agree this is an interesting question but have decided that a discussion on how to select the method for a triangulation is out of scope for this study and not the aim of our study. We have chosen the methods in the study due to: data availability (DID was possible because we have multiple time points) and the focus on diabetes research in which propensity score matching is the observational standard. Hence, we are comparing our application results to previously published results from PSM. (see: https://drc.bmj.com/content/8/1/e001238) **Comment 3:** “This opens up the possibility that treatment choice (or the extent of treatment received) and patient outcomes may be simultaneously predicted by common variables, which could bias the analysis because of a lack of balance across treatment groups, in contrast to the adherence affected RCT setting previously discussed.” The language “simultaneously predicted” is imprecise here. It could apply to M-bias type scenarios where there would not actually be confounding. **Response:** We clarified this: “This opens up the possibility that treatment choice (or the extent of treatment received) and patient outcomes may share a direct common cause which could bias the analysis because of a lack of balance across treatment groups, in contrast to the adherence affected RCT setting previously discussed.” **Comment 4:** “The IV approach addresses confounding by substituting each patients’ observed treatment with a predicted treatment. This prediction is made using a variable (the instrument) that is assumed to be independent of any confounders and only affects the outcome through the treatment.” This describes the mechanics of two stage least squares, which is just one of many estimators consistent for causal effects under IV assumptions. **Response:** We clarified this with: “The IV approach as commonly applied using Two-Stage Least Squares approach addresses confounding by substituting each patients’ observed treatment with a predicted treatment.” **Comment 5:** Randomization to a treatment group within a RCT is perhaps the best example of an IV”. Assuming randomization only impacts the outcome via received treatment, which is not always true. **Response:** we have changed this sentence to remove the wording “perfect IV” but have decided not to go into more detail. The additional provided literature (3 citations) add more context for the reader and the sentence is detailed enough to achieve an intuitive explanation of how IVs work.

 “Randomization to a treatment group within an RCT is often considered the strongest example of an instrumental variable, and is commonly used to adjust for non-adherence. [1,6,7]” **Comment 6:** “Examples of IVs for observational data include…” These are variables that past authors have asserted are IVs, but not necessarily valid IVs. Response: It was important to us to give readers without experience for IVs a feeling of what this variable could be. We are not claiming that these are valid IVs, just examples of variables that have been used as IVs from the literature. “Examples of variables which have been utilized as IVs for observational data include” **Comment 7:** The PERR approach seems also to have deep connections to Sofer et al (2016). **Response:** Thank you for pointing this paper out to us. **Comment 8:** In Figure 1, W0 has no direct effect on Y1 not through W1, and both W0 and W1 are completely independent of U. None of these things seem necessary for any of the methods to work. Also, Reviewer 1 noted that there is no Y0-->Y1 arrow and asked whether this is necessary for any results. I would note that for DiD the presence of such an arrow does indeed make the identifying assumptions less plausible because the direct association between U and Y0 through the U-->Y0 arrow needs to coincidentally equal the total association between U and Y1 both through the direct U-->Y1 arrow and the indirect U-->Y0-->Y1 path. This is discussed in Kim and Steiner (2021) **Response:** We address the data situation with Y_0à Y_1 in Section 4 scenario 2 as this is relevant to our diabetes application and because we also acknowledge that this could have an effect in the DID results. Besides this, we decided not to include a more complex data structure as we focused on the main assumptions of the IV and DID (PERR) method and believe that further complexity will take away from our main message. Thank you for pointing out Kim and Steiner (2021) to us. It seems that this explanation might relate to assumption DiD2. In our study DiD2 is not the main focus of simulation 1. **Comment 9:** “Complete adherence could be illustrated in Figure 1 by letting Z represent the randomized treatment assignment and removing all arrows into X from W0, W1 and U, so that only the Z → X arrow remains.”  Yes, but this wouldn’t fully capture it. You’d also need the Z-->X relationship to be deterministic. Response: We have clarified this “With a deterministic relationship Z→X, complete adherence could be illustrated in Figure 1 by letting Z represent the randomized treatment assignment and removing all arrows into X from W 0, W 1 and U, so that only the Z → X arrow remains.” **Comment 10:** “This could be estimated from fitting the following multivariable regression of Y1 on X and W1 as…” Under extra parametric modeling assumptions. **Comment 11:** The RHS of Equation (2) depends on W1 and is not the ATE except under strong effect homogeneity assumptions like in the linear model introduced after equation (2). **Response:** We have added a clarification to section 2 in response to reviewer 1 comments of similar nature. “Assuming throughout this section that the parametric model assumptions hold” **Comment 12:** The DiD section should also state the conditional parallel trends assumption (Abadie, 2005) as the assumption under which DiD is traditionally justified. Determining when parallel trends holds in terms of confounding is helpful and has been considered elsewhere. The proof of the sufficiency of DiD1 and DiD2 in Appendix 2 assumes linear models and is not general. Kim and Steiner (2021) should be cited concerning conditions under which parallel trends holds given linear models. Ghanem et al (2022) should be cited concerning more general structural conditions under which parallel trends holds. Sofer et al (2016) discuss how DiD can be justified under additive equi-confounding assumptions similar to your framing. **Response:** We have added clarification in the DiD assumptions section and mentioned Abadie, A. (2005). We have decided to refrain from giving this discussion too much space in our paper as our does not lie on the discussion and application of state-of-the-art DiD methodology. **Comment 13:** equation (6) is incorrect. It is missing an outer expectation over the covariates. Abadie (2005) provides singly robust estimators based on standardization or IPW, and Sant’anna and Zhao (2020) provide doubly robust ones. **Response:** we have corrected this **Comment 14:** (7) again makes strong parametric linear assumptions that aren’t explicitly stated and are not necessary. Maybe state at some point early on that the paper will consider simple parametric implementations of all methods to focus on the issue of triangulation. (I actually think that using DML with cross-fitting would put more focus on triangulation by removing nuisance model mis-specification as a factor.) **Response:** Please refer to response to comment 11. **Comment 15:** Finally, DiD estimates the effect of treatment on the treated (ATT), not the ATE. This should be stated, along with the extra assumption you’d require (as in the IV setting) that the ATT is equal to the ATE. **Response:** We have clarified this and added citation: Sant’Anna, Pedro HC, and Jun Zhao. "Doubly robust difference-in-differences estimators." Journal of econometrics 219.1 (2020): 101-122. **Comment 16:** The CF abbreviation is used before it’s introduced **Response:** Thank you, we have changed this **Comment 17:** Regarding binary outcomes, if the proportions in the treatment groups aren’t too close to 0 or 1 for parallel trends to make sense, it’s fine to just use a standard DiD estimator. (This is what you appear to have done in your simulations, judging from Table 1?) Otherwise, you might also want to mention the Universal DiD approach of Park and Tchetgen Tchetgen (2022). Also see the summary of other alternatives for DiD with binary outcomes therein. And it would be helpful if you cited the paper and assumptions justifying your suggested approach. Is it Wooldridge (2022) where parallel trends are now assumed on the logit scale? **Response:** Please refer to our response of comment 12. **Comment 18:** the DiD1 assumption which requires X to be independent of Y0" It just requires X to not be caused by Y0 **Response:** we have changed this in the text. **Comment 19:**  The framing of the POA-IV as a weakening of the assumptions required for DiD or IV is misleading. The method requires additional parametric assumptions in the form of the linear models (which should be explicitly stated as assumptions, including the point that Reviewer 1 made about the absence of the interaction in the outcome model being crucial) and also the assumption that U is not a cause of Y0. I would think it’s quite unusual for this latter assumption (that an unobserved confounder impacts the later measurement but not the earlier measurement of an outcome) to hold. Granted, the method seemed to have mild bias due to failure of this assumption in the simulations, but it’s not clear if this would generally be the case. It might be worth mentioning (if correct) that if there *is* unobserved confounding, POA and DiD would almost never both give the right answer since parallel trends requires a U-->Y0 arrow and POA requires its absence. This could potentially make it a particularly good method to include with DiD in a triangulation study? **Response:**  As Y0*Z is used as an IV the assumption that there is no direct pathway Y_0Z à Y_1 is directly addressed with the IV2 assumption. This assumption is not POA-IV specific but IV specific (please also see response to reviewer 1, comment 17) We highlight that the POA-IV will be beneficial in scenarios in which specific DiD and IV assumptions are violated: “Our simulations show that this approach is robust and leads to reliable results in scenarios in which key assumptions of the DiD and the IV approaches are violated, as long as the prior outcome-future treatment relationship does not suffer from unmeasured confounding”. We therefore also see the strength of including POA-IV and DiD together in a triangulation framework as they rely on a different set of assumptions which we have stressed in the section of the application study. Lastly, we cannot make any statements about the level of bias from our simulation study as this was not the focus of our study but agree that it will be interesting to investigate this in the future. **Comment 20:** The DAG in Figure 4 including an arrow from Y0*Z to X in addition to the arrows from Y0 and Z into X seems to reflect parametric assumptions and not really be a formal causal DAG, which is nonparametric and would not have the Y0*Z to X arrow since Y0*Z is a deterministic function of Y0 and Z. **Response:** Often no interactions are included in DAGs because it is a parametric assumption.  It has been suggested thought that it might add clarity to include interactions in DAGs ( https://academic.oup.com/ije/article/51/4/1047/6607680). Certainly in our case it helps to identify this interaction term as new IV and explain the POA-IV approach, which is why we made the decision to include it here.  We have included a clarification note under figure 4 with this citation. **Comment 21:** “Another approach for combining IV and DiD approaches has recently been proposed by Ye et al.58. The ‘instrumented DiD’ purports to offer robustness to time-varying unmeasured confounding and therefore offers considerable utility as an additional estimator within a triangulation analysis.” This is true, but they do assume a different data structure with treatments at two time-points. And again, I’d be interested in what are the criteria for determining level of “utility as an additional estimator within a triangulation analysis.” **Response:** The choice of the methods depends on the data available for treatment effect estimation. Given the data structure permits it, it is beneficial for triangulation to employ methods which use different parts of the data and employ different assumption as outlined in out study. Please refer to response to comment 2 regarding the choice of estimation methods for a specific triangulation analysis. 

# Reviewer report: Reviewer response for version 1

**DOI:** 10.21956/wellcomeopenres.25276.r118444

**Published:** 2025-02-27

**Authors:** Jeremy Labrecque

**Affiliations:** 1 Department of Epidemiology, Erasmus Universiteit Rotterdam, Rotterdam, South Holland, The Netherlands

I very much enjoyed reading this manuscript. More of this type of thinking needs to be done in triangulation. It is the importance of this type of work which led me to read it very carefully and provide many comments below, a few of which I think either need to be addressed or the authors should demonstrates why my reasoning is wrong. But again, I love the thinking that went into this manuscript and I hope my comments either help or it can easily be shown that my reasoning is wrong. I have put my more important comments in bold. 

 I have answered 'partly' to some of the questions that are asked of the reviewer not because there are many things missing, just that there are points that I think need to be addressed. 

**Comments:**

**Abstract and Methods:** This is a bit picky but for some reason I'm getting pickier with language as time goes by. I would say these methods circumvent confounding, it's that they do not require any assumptions related to exposure-outcome confounding.

**Section 1 Introduction: **

- **Paragraph 1:**

1) I would say that the double-blinded RCT with no loss to follow-up is the gold standard. An RCT can easily not be a gold standard.

 2) I would also not say "without the need to adjust for prognostic factors" rather I'd say without the need to adjust for confounders. In theory you never have to adjust for all prognostic factors, only those that are unbalanced.

 3) When you say it remains true even with non-adherence, are you referring to the ITT or the fact that you could use IV analysis? I think it would be nice to be clear for the reader here.

- **Paragraph 2:**

1) Technically confounders don't have to be common causes. They are any variables on a backdoor path that can be used to adjust away confounding. 

- **Paragraph 4:**

1) "This prediction is made using a variable (the instrument) that is assumed to be independent of any confounders and only affects the outcome through the treatment." An IV can be independent of exposure-outcome confounders and still be confounded with the outcome through other variables. I think it's more accurate to state that the IV must exchangeable with the outcome or that it must not be confounded with the outcome or related to it via selection bias. 

 2) " Because of this, IV analyses using observational data are generally equated with the creation of a pseudo-randomized controlled trial." I disagree with the wording. I wouldn't say it creates a pseudo-RCT because you aren't creating anything. You're making assumptions which may or may not hold. 

- **Paragraph 5:**

1) "The approach relies on the assumption of time invariant unmeasured confounding across both time periods." This could be stated more clearly. Are you saying that any unmeasured confounding must be assumed to be time invariant? Or that the unmeasured confounding remain constant over time? The way it is stated it sounds like you need to assume that unmeasured confounding exists. 

- **Figure 1:  There is no arrow from Y0 to Y1. Is that an assumption required for these methods? Because I think for many contexts, having a previous event does influence the probability of having a future event. But maybe in some contexts this is a reasonable assumption? And how about an arrow from W0 to Y1? That could also be possible. Are the results affected by the presence of such an arrow? I see that scenario 2 in the POA-IV simulation includes such an arrow. Could the authors either explain why this arrow is not included in any other simulation and/or whether they think such an arrow is implausible. I would think such an arrow is very plausible in many or most contexts. **

**Section 2:**

**2.1 **

- I'm not a fan of calling the assumption no unmeasured confounder because even if you've measured it you may not model it correctly. To me, it's more accurate to call it no uncontrolled confounding which fortunately has the same acronym,

**2.2 **

- "IV1: *Z* is associated, or predicts *X*;" is associated with
- The way you've listed the assumptions, isn't IV3 necessarily true if IV2 is true? If Z and Y1 share a common cause, then Z and Y1 will not be independent even conditional on W0, W1, X and U.                               

And if IV3 is exchangeability, as mentioned in the text, then you should say Z and Y1 do not share a common cause or are related through selection bias. 

**2.3**

- DiD2: In this case it is only conditional on W1 because W0 is not a confounder conditional on W0?

**2.5**

- **So this is probably my biggest comment. Have you chosen the right null hypothesis? Your null is that they're all targeting the same quantity but that's not what you want to reject. You want to reject the possibility that they are not targeting the same quantity. This point is too often overlooked in triangulation. If you fail to reject the null that they are targeting the same underlying quantity, that does not imply that they do. As is commonly repeated, failure to reject the null is not the same as accepting the null.**

**Section 3:**

**3.1 **

- I think the authors should mention the scale X and Y are generated on. For example, X was generated on the logic scale but Y was generated on the linear scale but by truncating values above 1 and below 0. These matter for section 4 believe. I'm also curious whether the truncation in generating Y matters. The more truncation occurs, the more that Y is not generated by the additive model you use.

**Section 4:**

- So I guess this is exploiting the fact that the interaction between Y0 and Z is an IV and therefore this method works even when Y0 and Z are not themselves IVs (which would make Y0*Z not an IV) because in adjusting for Y0 and Z, one eliminates the paths that violate the IV assumption for the Y0*Z IV. But this analysis relies on the assumption that there is a statistical interaction between Y0 and Z to generate X but no statistical interaction between Y0 and Z to generate Y1. Am I incorrect or should it be stated that there is no interaction between Y0 and Z to produce Y1 is also an assumption?
- One thing that should be noted is that interactions are scale specific. You've generated the data so that Y0*Z is not in the causal model for Y1 (although, if there is a lot of truncation it could be) on the additive scale but that means that if you estimated the outcome on the RR or OR scales, Y0*Z will cause the outcome, violating the IV assumption. My guess is that effects estimates on the RR or OR scales would be biased because if there must be an interaction between Y0*Z on the multiplicative scale if there is none on the additive scale. Or maybe there's something I'm missing here.

**Section 5:**

- **Paragraph 2:**

1) The first sentence states the question in non-causal language (in that the risk could be estimated in each group without causal assumption). Given the clear causal goal here, I would suggest stating the question in causal language. 

- **Paragraph 6:**

1) Why use matching and not IPTW? Matching changes the estimand to one in the matched population, not the full population whereas IPTW estimates the effect in the full population. 

 2) " *Y*_0_ was included as a measured confounder in all models for *Y*_1_ as it has been found in previous studies that prior infections are associated with the risk of experiencing an infection in the study period" So in your application, Y0 is assumed to have an affect on Y1. Another argument to include it in more of your simulations. 

 5.2: You say the POA-IV results are poor because of the high uncertainty. I would say poor is not the right word here as I would normally understand that as referring to validity rather than uncertainty due to random error. 

**Table 8: So here I have a problem. When you fail to reject the null, you don't get to say you accept the null. Just that you weren't able to reject the null. Similarly, if you fail to reject the null that they're similar, you cannot imply that they're similar, only that you've failed to reject that null. It could be that you're underpowered. Again, this situation is more like an equivalence test where we design a test to reject the hypothesis that the estimate is outside some region (chosen by the user) that is considered null. Triangulation wants evidence that estimates are similar, not evidence that they're different.**

**A clear example of the issue here is the first two rows of Table 8. The difference in the effect estimate between CaT and PSM (0.73) is very, very similar to the difference between IV and CF (0.71). Yet in the first case you conclude they're not similar and in the second case you conclude that they are similar. This decision is being driven by the uncertainty in the estimates. This increased uncertainty is not making the estimates more similar, it's making it more difficult to reject the null hypothesis!** **This problem occurs entirely because a non-significant test is being misconstrued as evidence for the null.**

**This is really important in triangulation because I don't want to identify pairs of estimates that are "sufficiently similar" as identified by a hypothesis test that is designed to reject the null that they're similar. Any failure to reject the null could be because the null is true or because there is too much noise to reject the null. What I want in triangulation is evidence that they are similar, not evidence that they are not dissimilar which is what the test is giving you.**

Is the work clearly and accurately presented and does it cite the current literature?

Yes

If applicable, is the statistical analysis and its interpretation appropriate?

Partly

Are all the source data underlying the results available to ensure full reproducibility?

Yes

Is the study design appropriate and is the work technically sound?

Partly

Are the conclusions drawn adequately supported by the results?

Partly

Are sufficient details of methods and analysis provided to allow replication by others?

Yes

Reviewer Expertise:

Epidemiology methods and causal inference

I confirm that I have read this submission and believe that I have an appropriate level of expertise to confirm that it is of an acceptable scientific standard, however I have significant reservations, as outlined above.

# Response

**Published:** 2025-07-31

**Authors:** Laura Güdemann

**Affiliations:** Faculty of Health and Life Sciences, University of Exeter, Exeter, England, UK

**Thank you for your helpful suggestions and encouraging feedback, which guided our revisions in a meaningful way.** **Comment 1:** This is a bit picky but for some reason I'm getting pickier with language as time goes by. I would say these methods circumvent confounding, it's that they do not require any assumptions related to exposure-outcome confounding. **Answer:** We agree that the IV does not require assumptions related to exposure-outcome confounding but the DiD approach requires the assumption that effect of confounders is constant over time. We still tried to accommodate this comment by changing the sentence to: “Causal inference methods such as Instrumental Variable and Prior Event Rate Ratio approaches enable the estimation of causal effects even in the presence of unmeasured or imperfectly measured confounding factors.” **Comment 2:** I would say that the double-blinded RCT with no loss to follow-up is the gold standard. An RCT can easily not be a gold standard. **Answer:** We agree and changed this to: “The gold standard approach for evaluating the efficacy of treatments is a double-blinded randomized controlled trial (RCT) with no loss to follow-up.” **Comment 3:** I would also not say "without the need to adjust for prognostic factors" rather I'd say without the need to adjust for confounders. In theory you never have to adjust for all prognostic factors, only those that are unbalanced. **Answer:** We changed this to: “…without the need to adjust for confounders…” **Comment 4:** When you say it remains true even with non-adherence, are you referring to the ITT or the fact that you could use IV analysis? I think it would be nice to be clear for the reader here. **Answer: **Due to the addition we made in response to comment 3 we have decided to delete this sentence as it does not add relevant information. **Comment 5:** Technically confounders don't have to be common causes. They are any variables on a backdoor path that can be used to adjust away confounding. **Answer:** As we are defining confounding and not confounders in this section we don’t think we need to add much to satisfy your comment. We have added an additional sentence to add a generalization and have added two citations from Pearl 1993, and Pearl 2000 for the back-door path criterion.

 “More generally, variables which are not necessarily common causes can be confounders, if they can be used to block the back door-path from the treatment decision and the outcome of interest.” **Comment 6:** "This prediction is made using a variable (the instrument) that is assumed to be independent of any confounders and only affects the outcome through the treatment." An IV can be independent of exposure-outcome confounders and still be confounded with the outcome through other variables. I think it's more accurate to state that the IV must exchangeable with the outcome or that it must not be confounded with the outcome or related to it via selection bias. **Answer:** We changed the sentence to: “This prediction is made using a variable (the instrument) that is assumed to be unconfounded with the outcome and only affects the outcome through the treatment.” **Comment 7:** " Because of this, IV analyses using observational data are generally equated with the creation of a pseudo-randomized controlled trial." I disagree with the wording. I wouldn't say it creates a pseudo-RCT because you aren't creating anything. You're making assumptions which may or may not hold. **Answer:** We agree that the IV assumptions are at the heart of the method but also acknowledge that the phrase “pseudo-randomization” refers to the utilization of *X*   in the outcome model of the TSLS. We like the comparison of the IV method with the RCT (how it is structured in the paper) and the explanation of randomization in an RCT being and IV. We believe that this makes it easier to understand the intuition of the IV approach. This terminology is also frequently used in IV explanations.

 See for example: https://www.jstage.jst.go.jp/article/ace/2/3/2_69/_pdf https://onlinelibrary.wiley.com/doi/pdf/10.1002/hec.3861 **Comment 8:** "The approach relies on the assumption of time invariant unmeasured confounding across both time periods." This could be stated more clearly. Are you saying that any unmeasured confounding must be assumed to be time invariant? Or that the unmeasured confounding remain constant over time? The way it is stated it sounds like you need to assume that unmeasured confounding exists. **Answer:** We clarified this and changed the sentence to: “The approach relies on the assumption that the effect of unmeasured confounding on the outcome is constant across both time periods.” **Comment 9:** There is no arrow from Y0 to Y1. Is that an assumption required for these methods? Because I think for many contexts, having a previous event does influence the probability of having a future event. But maybe in some contexts this is a reasonable assumption? And how about an arrow from W0 to Y1? That could also be possible. Are the results affected by the presence of such an arrow? I see that scenario 2 in the POA-IV simulation includes such an arrow. Could the authors either explain why this arrow is not included in any other simulation and/or whether they think such an arrow is implausible. I would think such an arrow is very plausible in many or most contexts. **Response:  **We start the introduction of the paper with the standard assumptions made for PERR and IV (explanation in method section). In section 4 scenario 2 we investigate what will happen to results of the DID (related to PERR approach) and explain situations in which a Y_0àY_1 relationship is reasonable.  We felt that the relationship between W_0—> Y_1 is out of the scope of this paper especially because we acknowledge a relationship between W_0àW_1àY_1 which is much more important, especially given the diabetes application study. Please also note, that in this study the set of variables for W_0 and W_1 are the same for simplicity. We feel it will not add to the overall message of our paper if we introduce more complexity. **Comment 10:** I'm not a fan of calling the assumption no unmeasured confounder because even if you've measured it you may not model it correctly. To me, it's more accurate to call it no uncontrolled confounding which fortunately has the same acronym. **Response:** The terminology “no unmeasured confounding” is frequently used and in alignment with the focus of our paper. (Eg.: Miguel, A., ROBINS HERNAN, and M. James. Causal inference: what if. CRC PRESS, 2023.) But, we can see where you are coming from and agree that these are two important aspects.

1. All the data we need to adjust is measured
2. We use the data that we have correctly/ no model misspecifications.

Part of this is already included in the sentence “If all confounding factors are known and can be appropriately measured and adjusted for”. We also added a sentence to clarify that we are making parametric model assumptions: “Assuming throughout this section that the parametric model assumptions hold, this could be estimated from fitting the following multivariable regression of Y 1 on X and W 1 as… ” **Comment 11:** "IV1: *Z* is associated, or predicts *X*;" is associated with **Response:** we changed it to “IV1: Z is associated with, or predicts X” **Comment 12:** The way you've listed the assumptions, isn't IV3 necessarily true if IV2 is true? If Z and Y1 share a common cause, then Z and Y1 will not be independent even conditional on W0, W1, X and U. **Comment 13:** And if IV3 is exchangeability, as mentioned in the text, then you should say Z and Y1 do not share a common cause or are related through selection bias. **Response: **We can think of a situation where IV2 does not imply IV3 (see below where U_2 is a common cause). To be very clear we also included further explanation on IV2: “Assumption IV2 is also referred to as the exclusion restriction and requires Z to only influence Y1 through X but not directly”  **Comment 14:** DiD2: In this case it is only conditional on W1 because W0 is not a confounder conditional on W0? **Response:** in the DiD framework Y_0 is an outcome too therefore W_0 is indeed a confounder for Y_0. **Comment 15:** So this is probably my biggest comment. Have you chosen the right null hypothesis? Your null is that they're all targeting the same quantity but that's not what you want to reject. You want to reject the possibility that they are not targeting the same quantity. This point is too often overlooked in triangulation. If you fail to reject the null that they are targeting the same underlying quantity, that does not imply that they do. As is commonly repeated, failure to reject the null is not the same as accepting the null. **Response:** We have made several changes in the text to address your comment and comment 23 below regarding our wording in table 8 and the aim of the test. We also added the important clarification that if we fail to reject the H0, it does not mean that we assume it is true.  We have added: “The measure assesses whether the observed variability in effect sizes across methods is greater than what would be expected by chance alone. Rejecting the null hypothesis of similarity will provide evidence that the measures do not address the same underlying quantity. This is important knowledge when triangulating results from different methods and when interpreting differences in estimated effect sized. On the other hand, if the null hypothesis is not rejected, we can not conclude that the measured are similar as the analysis could be underpowered to detect dissimilarities. ” We have also adjusted the interpretation of the test result in section 3 and 5 to ensure that we do not interpret “not rejecting H0” as sure evidence for similarity. **Comment 16:** I think the authors should mention the scale X and Y are generated on. For example, X was generated on the logic scale but Y was generated on the linear scale but by truncating values above 1 and below 0. These matter for section 4 believe. I'm also curious whether the truncation in generating Y matters. The more truncation occurs, the more that Y is not generated by the additive model you use. **Response:** Thank you for looking so closely into our code, this gives us confidence that our work/code can be reproduced/ understood by others! We went back to the code and checked if the truncation of Y made a difference. We can confirm that this part of the code is only a safety step to ensure all values of Ye0 and Ye1 are between 0 and 1 and was actually not needed in our final version of the simulation published. We run all the simulations again without the truncation as due to our parameter value choices we don’t need to restrict the value of Yeo and Ye1. For the simulation results published, essentially no values were truncated. We have added a note in the R code for clarification. A detailed description of how the data was generated can be found in the appendix which we published here: https://github.com/GuedemannLaura/POA-IV as per journal requirements. **Comment 17:  **So I guess this is exploiting the fact that the interaction between Y0 and Z is an IV and therefore this method works even when Y0 and Z are not themselves IVs (which would make Y0*Z not an IV) because in adjusting for Y0 and Z, one eliminates the paths that violate the IV assumption for the Y0*Z IV. But this analysis relies on the assumption that there is a statistical interaction between Y0 and Z to generate X but no statistical interaction between Y0 and Z to generate Y1. Am I incorrect or should it be stated that there is no interaction between Y0 and Z to produce Y1 is also an assumption? **Response:** It is correct that the method relies on the assumption that there is no direct path Y_0Z à Y_1.  Z is not a suitable IV in this context anymore because of YàY_1 (violation of IV2). We show that we can create a new IV with the interaction term Y_0*Z. IV2 must therefore also be assumed for Y_0*Z. **Comment 18:** One thing that should be noted is that interactions are scale specific. You've generated the data so that Y0*Z is not in the causal model for Y1 (although, if there is a lot of truncation it could be) on the additive scale but that means that if you estimated the outcome on the RR or OR scales, Y0*Z will cause the outcome, violating the IV assumption. My guess is that effects estimates on the RR or OR scales would be biased because if there must be an interaction between Y0*Z on the multiplicative scale if there is none on the additive scale. Or maybe there's something I'm missing here. **Response: **As discussed in our response to Comment 16, there is no truncation in our setup. We explicitly specified the data-generating process so that there is no causal path from the interaction term Y0×Z to Y1​. While it is true that statistical interactions may appear on the multiplicative scale (e.g., risk ratios or odds ratios), these are artifacts of the nonlinear transformation and do not represent actual causal pathways. As such, they do not violate the IV exclusion restriction, which depends on the causal structure, not the statistical scale. Our simulation results confirm this: we observe bias only in scenarios where the IV assumptions are explicitly violated, and not when interactions appear solely as statistical artifacts on transformed scales. **Comment 19:** The first sentence states the question in non-causal language (in that the risk could be estimated in each group without causal assumption). Given the clear causal goal here, I would suggest stating the question in causal language. **Response:** We changed the text to  “We used routine data from Clinical Practice Research Datalink from 2016-2019 to examine if SGLT2i (n = 1966) initiation as second-line treatment after MFN causes a relative increase of genital infection risk compared to DPP4i (n = 4033) initiation.” **Comment 20:** Why use matching and not IPTW? Matching changes the estimand to one in the matched population, not the full population whereas IPTW estimates the effect in the full population. **Response:**  Because previous published work we are comparing our results with have used propensity score matching (see: https://drc.bmj.com/content/8/1/e001238).  Comparison of our research findings with previous work is added value to important research question in T2D research and was therefore a priority. **Comment 21: **"Y0 was included as a measured confounder in all models for Y1 as it has been found in previous studies that prior infections are associated with the risk of experiencing an infection in the study period" So in your application, Y0 is assumed to have an affect on Y1. Another argument to include it in more of your simulations. **Response:**  We look at this assumption explicitly in Scenario 2 of the second simulation and explain when this simulation is reasonable in in real data. For the first simulation we have decided to leave this aspect out to reduce complexity and focus on introducing the main data structure concepts of the causal inference methods. We agree that our data structure choice is not the only way of how the data could have been simulated, but we believe adding more complexity to the data structure will not change the main take aways of the first simulation. **Comment 22:** (5.2) You say the POA-IV results are poor because of the high uncertainty. I would say poor is not the right word here as I would normally understand that as referring to validity rather than uncertainty due to random error. **Response:** we see your point and have taken this out. **Comment 23:** So here I have a problem. When you fail to reject the null, you don't get to say you accept the null. Just that you weren't able to reject the null. Similarly, if you fail to reject the null that they're similar, you cannot imply that they're similar, only that you've failed to reject that null. It could be that you're underpowered. Again, this situation is more like an equivalence test where we design a test to reject the hypothesis that the estimate is outside some region (chosen by the user) that is considered null. Triangulation wants evidence that estimates are similar, not evidence that they're different.

 A clear example of the issue here is the first two rows of Table 8. The difference in the effect estimate between CaT and PSM (0.73) is very, very similar to the difference between IV and CF (0.71). Yet in the first case you conclude they're not similar and in the second case you conclude that they are similar. This decision is being driven by the uncertainty in the estimates. This increased uncertainty is not making the estimates more similar, it's making it more difficult to reject the null hypothesis! This problem occurs entirely because a non-significant test is being misconstrued as evidence for the null.

 This is really important in triangulation because I don't want to identify pairs of estimates that are "sufficiently similar" as identified by a hypothesis test that is designed to reject the null that they're similar. Any failure to reject the null could be because the null is true or because there is too much noise to reject the null. What I want in triangulation is evidence that they are similar, not evidence that they are not dissimilar which is what the test is giving you. **Response:** We have addressed this comment together with comment 15 and therefore refer to our response to comment 15.
